# Conformational flexibility driving charge-selective substrate translocation across a bacterial transporter†

**DOI:** 10.1039/d4sc00345d

**Published:** 2024-05-13

**Authors:** Devika Vikraman, Bibhab Bandhu Majumdar, Sharavanakkumar SK, Conrad Weichbrodt, Niels Fertig, Mathias Winterhalter, Jagannath Mondal, Kozhinjampara R. Mahendran

**Affiliations:** a Transdisciplinary Research Program, Rajiv Gandhi Centre for Biotechnology Thiruvananthapuram 695014 India mahendran@rgcb.res.in; b Manipal Academy of Higher Education Manipal Karnataka-576104 India; c School of Advanced Sciences, VIT-AP University Amaravati Andhra Pradesh 522237 India; d Nanion Technologies GmbH Munich 80339 Germany; e School of Science, Constructor University Campus Ring 1 28759 Bremen Germany; f Center for Hybrid Nanostructures (CHyN), Universität Hamburg Luruper Chaussee 149 Hamburg 22761 Germany; g Tata Institute of Fundamental Research Hyderabad Telangana-500046 India jmondal@tifrh.res.in

## Abstract

Bacterial membrane porins facilitate the translocation of small molecules while restricting large molecules, and this mechanism remains elusive at the molecular level. Here, we investigate the selective uptake of large cyclic sugars across an unusual passive membrane transporter, CymA, comprising a charged zone and a constricting N terminus segment. Using a combination of electrical recordings, protein mutagenesis and molecular dynamics simulations, we establish substrate translocation across CymA governed by the electrostatic pore properties and conformational dynamics of the constriction segment. Notably, we show that the variation in pH of the environment resulted in reversible modulation of the substrate binding site in the pore, thereby regulating charge-selective transport of cationic, anionic and neutral cyclic sugars. The quantitative kinetics of cyclic sugar translocation across CymA obtained in electrical recordings at different pHs are comparable with molecular dynamics simulations that revealed the transport pathway, energetics and favorable affinity sites in the pore for substrate binding. We further define the molecular basis of cyclic sugar translocation and establish that the constriction segment is flexible and can reside inside or outside the pore, regulating substrate translocation distinct from the ligand-gated transport mechanism. Our study provides novel insights into energy-independent large molecular membrane transport for targeted drug design strategies.

## Abstract 2

Here, we establish the conformational dynamics of a specialized natural passive bacterial transporter, CymA, using single-channel recordings and molecular dynamics simulations.

## Graphical abstract

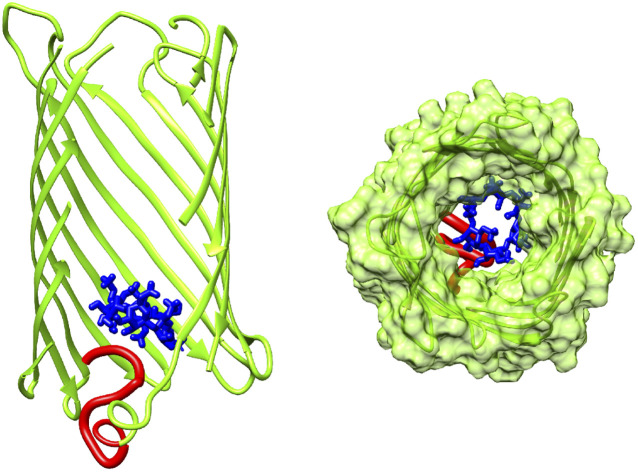

## Introduction

The bacterial outer membrane serves as a permeability barrier and contains stable β-barrel pores for shuttling ions and substrates.^1–3^ While several passive channels are present for the diffusion of small molecules below 600 Da, the uptake of bulky substrates is mediated through energy-dependent transporters.^1–4^ The unique structural architecture of outer membrane porins containing constriction regions facilitates the transport of small molecules and makes them attractive targets for sensing and sequencing applications.^5–13^ Interestingly, several porins consist of extracellular and periplasmic loops whose functional significance associated with molecular transport has not been precisely demonstrated.^2,12,14^ Some membrane pores, such as substrate-specific porins, contain a critical sensing zone that acts as a binding site promoting selective molecular translocation.^5,8,15–18^ Although high-resolution structures are available, their molecular basis of selectivity remains underexplored.^1,3,15–18^

Here, we investigate molecular transport across a specialized sugar-specific porin, CymA, derived from the outer membrane of the Gram-negative bacteria *Klebsiella oxytoca*.^17,19–23^ The structure of CymA revealed a monomeric beta-barrel fold consisting of densely packed charged residues, acting as an affinity site for alpha-cyclodextrin (αCD) binding.^17^ Specifically, the abundantly present negatively charged residues at the pore surface render the pore cation-selective, which has been exploited for the single-molecule sensing of various cyclodextrins.^22,23^ Remarkably, previous structural studies have revealed an unusual architecture of this pore wherein a 21-residue N terminus is folded into the pore lumen from the periplasmic side, forming a plug and restricting its diameter.^4,17^ Notably, only the first nine residues of the N terminus could be observed in the structure and previous reports suggest that this segment is ejected from the pore during the translocation of αCD, yet experimental evidence is missing.^17^ Although its structural conformation is similar to that of active transporters due to the presence of the N terminal plug, CymA functions as a passive transporter.^4,9,17,24^ Also, CymA showed a distinct structural geometry and functionality compared to most trimeric porins, consisting of several extracellular and periplasmic loops.^2,3,5^ Therefore, establishing the clear functional role of the N terminus and electrostatic pore properties in mediating the transport of large substrates across CymA is vital.

Most previous pH studies on membrane porins have focused on gating.^25,26^ In this study, using high-resolution single-channel electrical recordings, we elucidate the transport kinetics of cyclic sugars at different pH conditions through native and truncated CymA (ΔN15), in which the first 15 amino acid residues in the N terminus have been removed. Here, we propose that the dense charge pattern in the pore can be modulated by pH for effective substrate transport. Molecular dynamics (MD) simulations revealed the entry and exit of cyclic sugars through the pores at a microscopic scale and associated free energetics. We show that the pH modulates the selective transport of cyclic sugars through CymA owing to its specific charge pattern and the constriction segment induces conformational changes controlling transport. Importantly, we show that this new class of natural pores can be used for applications in nanobiotechnology and nanopore chemistry.

## Results and discussion

### Effect of pH on the structural and electrical properties of the CymA pores

**Fig. 1:**
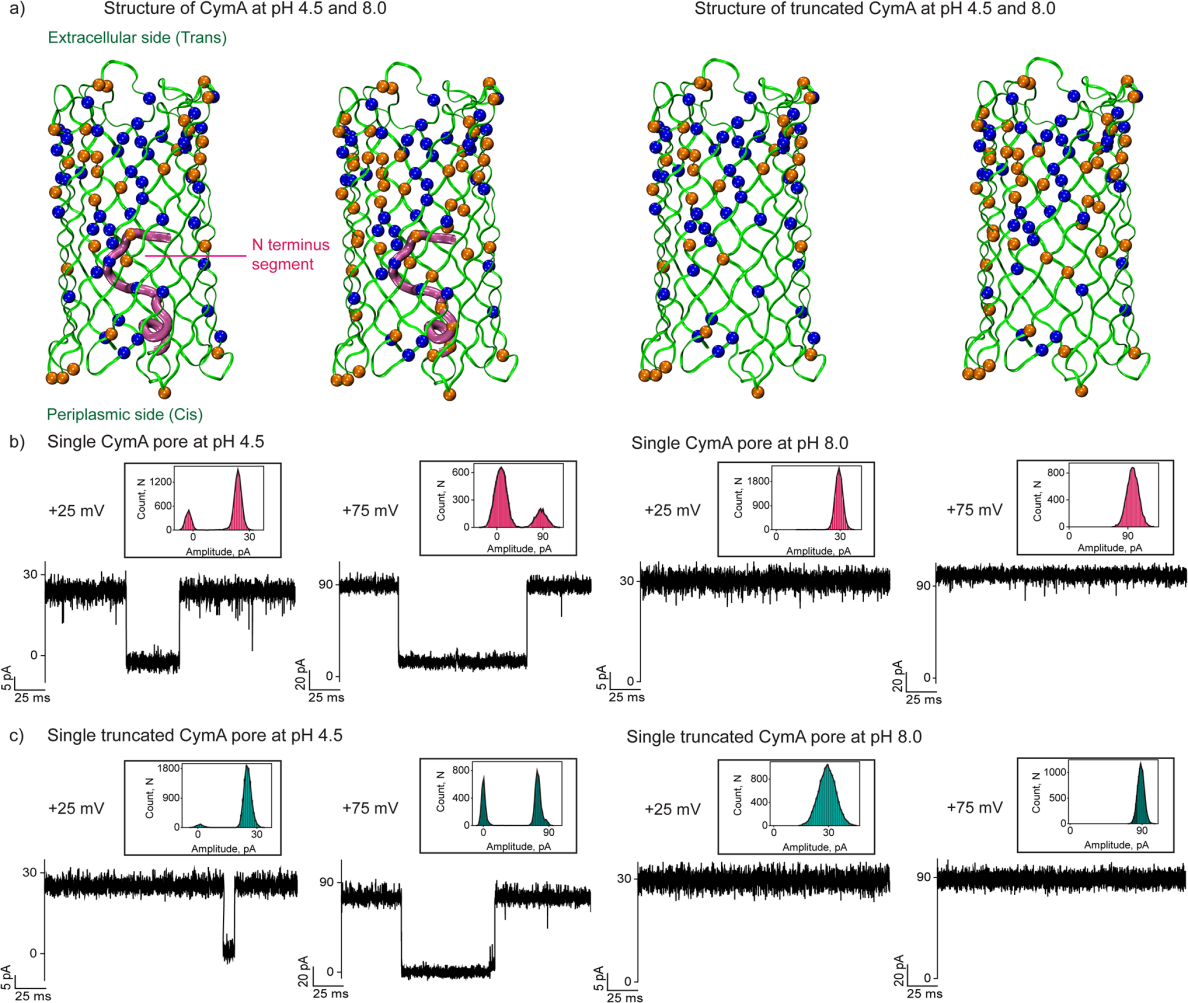
Structure and pH effect on single-channel properties of CymA pores. (a) Structure of native CymA with the N terminus in the pore at pH 4.5 (charge +14) and pH 8.0 (charge −6). Native CymA is shown in green ribbon representation (residues 16–324) and residues 1–15 in magenta tube representation (N terminus). Structure of truncated CymA (deletions 1–15) without the N terminus at pH 4.5 (charge +15) and at pH 8.0 (charge −4). Truncated CymA is shown in green ribbon representation. The negatively charged residues are shown as orange space-filling spheres and positively charged residues are shown in blue space-filling spheres. The extracellular side consists of dense negatively charged residues compared to the periplasmic side of the pore. (b) Electrical recordings of single native CymA at pH 4.5 and 8.0 at +25 mV and +75 mV. (c) Electrical recordings of single truncated CymA at pH 4.5 and 8.0 at +25 mV and +75 mV. The corresponding current amplitude histogram obtained by fitting the distribution to a Gaussian is shown in the inset. Electrolyte: 1 M KCl, 10 mM citrate for pH 4.5 and 10 mM HEPES for pH 8.0. The current signals were digitally filtered at 7 kHz.

The missing residues (residues 10 to 21) in the native CymA (PDB: 4D51) were added using the CHARMM-GUI web server.^27^ The PDB reader and modeler plugin of the CHARMM-GUI web server employ the GalaxyFill algorithm to add the missing residues.^28^ The CymA includes many ionizable amino-acid residues, including glutamic acid, aspartic acid, lysine, arginine and histidine spread across the protein (Fig. 1a and S1, Table S1 and ESI text†). Considering the presence of multiple ionizable residues with a heterogeneous environment inside the protein, we employed a widely recognized p*K*_a_-assignment program, PDB2PQR, that uses the PropKa program to obtain a measure of the p*K*_a_ of each of the ionizable residues inside the protein.^29,30^ Depending on the local environment, the p*K*_a_ of a subset of the ionizable residues would be modulated from its respective model p*K*_a_ value (*i.e.* isolated amino acid in water) (Table S1†). Accordingly, at pH 4.5, we found that a subset of the ionizable amino acid residues would remain protonated and a subset would be deprotonated depending on their respective p*K*_a_. Based on this, the native CymA's net charge was determined to be +14 at pH 4.5, while the same was −6 at pH 8.0. Truncated CymA charges were determined to be +15 at pH 4.5 and charge −4 at pH 8.0 (Fig. 1a).

We reconstituted CymA proteins into 1,2-diphytanoyl-3-*sn*-phosphatidylcholine (DPhPC) lipid bilayers in 1 M KCl with acidic (pH 4.5) and basic (pH 8.0) buffers and characterized single-channel properties using electrical recordings. Insertion of native CymA into the membrane was facilitated at a high voltage of +200 mV at pH 4.5, resulting in pore formation and the unitary conductance histogram was obtained based on multiple single-channel insertions (*n* = 25). The pore showed a mean unitary conductance (*G*) of 0.95 ± 0.12 nS at +50 mV and 0.85 ± 0.12 nS at −50 mV (Fig. 1b and S2†). The pore showed significant gating above ±75 mV and the gating frequency increased at negative voltages. In pH 8.0 buffer, native CymA rapidly inserted into lipid bilayers and formed pores that showed an increased mean unitary conductance (*G*) of 1.25 ± 0.12 nS at +50 mV and 1.13 ± 0.12 nS at −50 mV (*n* = 25). Notably, the gating of the pore significantly reduced and the pore remained in the stable open state up to ±100 mV (Fig. 1b). Next, we characterized the single channel properties of truncated CymA at different pH conditions (4.5 and 8.0) (Fig. 1c and S3†). Truncated CymA inserted into lipid bilayers and formed pores with a mean unitary conductance (*G*) and gating properties similar to native CymA (Fig. 1c and S3†). The statistical analysis of 25 different CymA pores (native and truncated CymA) revealed an asymmetry in the ion conductance and gating pattern with the voltage polarity (Fig. 1, S2 and S3†). For example, pores showed slightly higher conductance at positive voltages and more frequent gating at negative voltages. Based on this asymmetry in the pore conductance and gating with the direction of the voltage, we presumed the pore orientation, as shown in our previous studies^22,31^ (Fig. 1 and S1–S3†). Furthermore, the structure of CymA revealed that the extracellular side of the pore is associated with densely packed negatively charged residues and the periplasmic side of the pore is associated with significantly less negatively charged residues.^17^ Previously, we have shown that cationic CDs and peptides bind to the *trans* side of the pore with high affinity compared to the *cis* side, which agrees with the charge distribution in the pore, which allowed us to presume pore orientation.^22,31^ Thus, adding CymA to the *cis* side of the preformed bilayer results in pore insertion with the extracellular side (pore entrance and dense charge pattern) exposed at the *trans* compartment (Fig. 1a and S1†).

### Interaction of cationic cyclodextrin with native CymA at different pHs

**Fig. 2:**
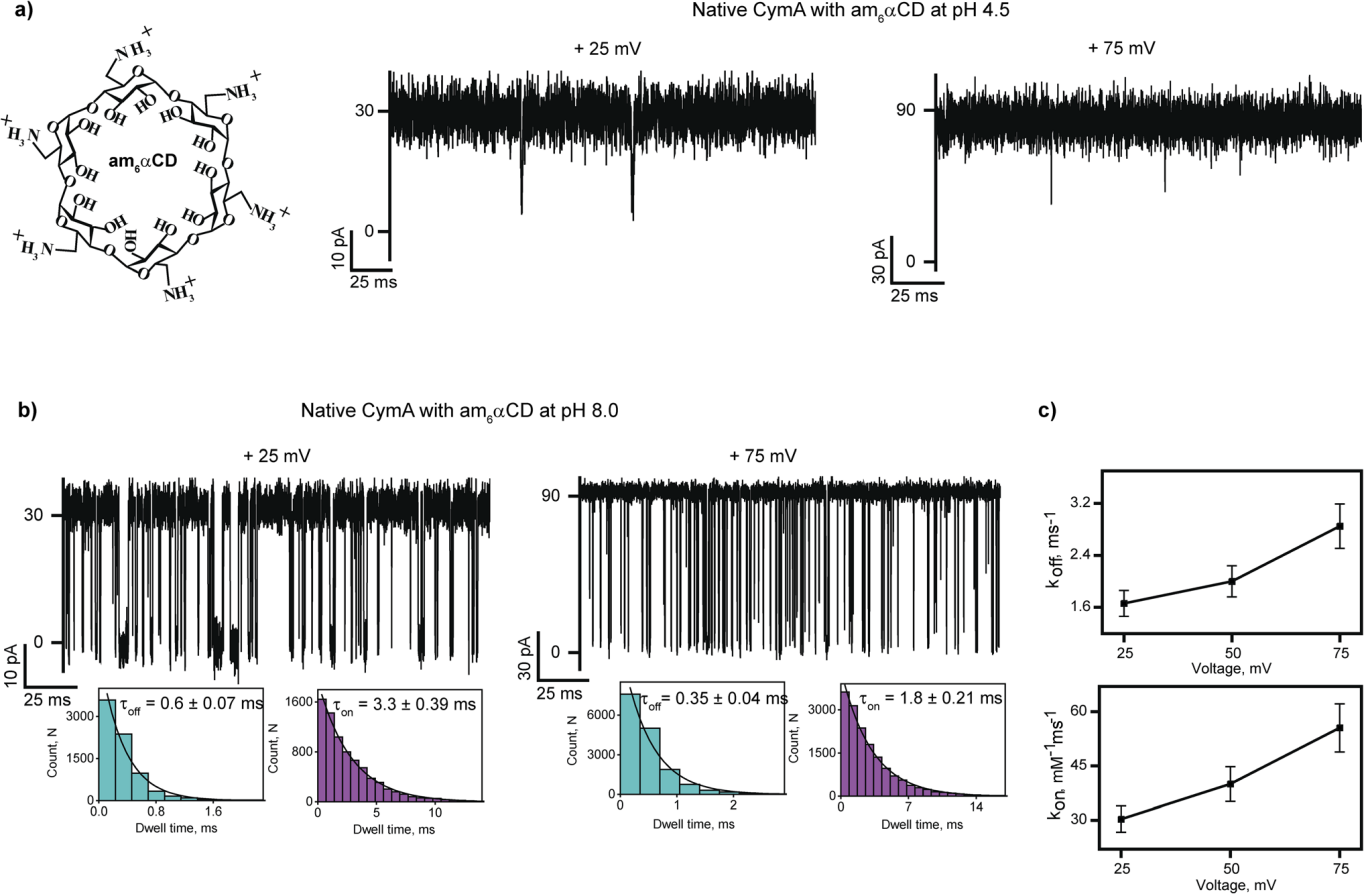
Interaction of am_6_αCD with native CymA. (a) Chemical structure of am_6_αCD and electrical recordings of single native CymA in the presence of 10 μM am_6_αCD added to the *trans* side at +25 mV and +75 mV at pH 4.5. (b) Electrical recordings of single native CymA in the presence of 10 μM am_6_αCD added to the *trans* side at +25 mV and +75 mV at pH 8.0. Insets show the *τ*_off_ and *τ*_on_ dwell time histograms of CD blocking fitted with a monoexponential probability function. (c) A plot of *k*_on_ and *k*_off_*versus* the applied voltage for am_6_αCD binding at pH 8 is shown. Mean values (±s.d.) from three different experiments are shown. Electrolyte: 1 M KCl, 10 mM citrate for pH 4.5 and 10 mM HEPES for pH 8.0. The current signals were digitally filtered at 7 kHz.

Next, we investigated the interaction of cationic cyclodextrin, am_6_αCD, with native CymA at pH 4.5 and 8.0 (Fig. 2 and S4†). At first, the binding of cationic am_6_αCD with native CymA was examined with the pores that exhibited minimal gating at different voltages at pH 4.5 as acidic pH promoted channel closure. The addition of 10 μM am_6_αCD to the extracellular side (*trans*) of native CymA produced negligible ion current blockages at different voltages (Fig. 2a and S4†). Consequently, the binding kinetics to elucidate translocation could not be determined.

Next, the interaction of am_6_αCD with native CymA was investigated at pH 8.0, where the pore remained in the open state. The addition of 10 μM am_6_αCD to the *trans* side of native CymA produced well-defined ion current blockages at specifically positive voltages from +25 mV to +100 mV, indicating electrophoretic pulling of the CDs into the pore promoting electrostatic binding with negatively charged pore residues (Fig. 2a and b, S1 and S4†). Notably, negligible ion current blockages were observed at negative voltages on the *trans* side addition of am_6_αCD, indicating that the applied voltage provides the driving force to pull the charged CDs into the pore. This voltage-dependent time-resolved ion current blockages were analyzed further to distinguish binding and translocation of am_6_αCD as previously demonstrated for various charged analytes and nanopores.^22,32,33^ Notably, the mean dwell times of am_6_αCD blockages (*τ*_off_) were determined to be 0.6 ± 0.07 ms (*n* = 3) at +25 mV and 0.35 ± 0.04 ms (*n* = 3) at +75 mV. The dissociation rate (*k*_off_ = 1/*τ*_off_) increased with increasing positive voltages, indicating the successful translocation of the CDs across the pores in agreement with an increase in the association rate (*k*_on_) (Fig. 2b and c and S4†). Importantly, am_6_αCD binds to native CymA with high affinity (*K*_D_) at pH 8.0 and this binding affinity facilitates the translocation of am_6_αCD based on the side of the CD addition and the direction of the applied voltage (Fig. 2b and c and Table S2†).

**Fig. 3:**
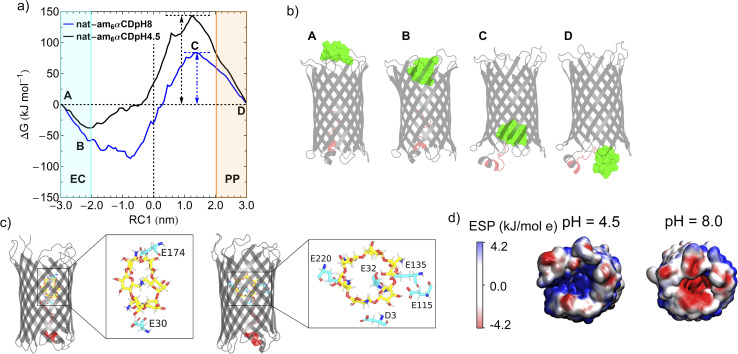
Molecular dynamics simulations and free energy profile of am_6_αCD translocation. (a) One-dimensional free energy profiles of am_6_αCD translocation at pH 8 (blue curve) and 4.5 (black curve). Multiple locations of the free energy profile are labeled from A to D, referring to various stages of the CD translocation events. EC and PP refer to the extracellular and periplasmic regions, respectively. (b) Snapshots of the pore model showing various stages of the CD translocation process. The CD is shown as green space-filling spheres. The pore is shown in cartoon representation (gray). The N terminus (residues 1–15) is shown in red. (c) Snapshots of am_6_αCD translocation at pH 4.5 and 8.0 showing the acidic amino acid residues involved in hydrogen bonding interactions with CD. The CD and amino acid residues are shown in stick representation. (d) Electrostatic potential distribution map (kJ mol^−1^ e) of the native CymA surface at pH 4.5 and 8.0.

To investigate the molecular details of am_6_αCD translocation across native CymA, we performed molecular dynamics (MD) simulations at pH 4.5 and 8.0 (Fig. 3). For a quantitative measure of the location of the free energy basin and potential barrier during CD translocation across the channel, we employed a well-recognized enhanced sampling simulation approach called umbrella sampling simulation.^34^ To this end, the distance vector between the centers of mass of the protein and the CD along the *z*-axis was chosen as the reaction coordinate (RC1). First, am_6_αCD was inserted in the hydrated membrane-protein system and aligned along the *z*-axis (Fig. S5†). Then, to generate initial configurations for umbrella sampling, steered molecular dynamics (MD) simulations were performed by prompting am_6_αCD to translocate through the pore from the extracellular to the periplasmic side. Am_6_αCD adopted diverse orientations during its translocation through CymA, providing discrete sets of initial configurations along RC1 ranging from −3.0 nm to +3.0 nm (Fig. S6†). The negative and positive values of the reaction coordinate refer to the CD translocating from the extracellular region toward the periplasmic region. As a reference, an RC1 value of 0 nm refers to the CD residing very close to the center of the protein. We obtained a free energy barrier of approximately 145 kJ mol^−1^ for the am_6_αCD translocation through native CymA at pH 4.5 compared to a significantly smaller free energy barrier of about 80 kJ mol^−1^ at pH 8.0 (Fig. 3a).

The free energy barrier is computed as the difference in free energy between the maximum energy height and the extracellular entrance to the CymA.^3,35^ The error bars associated with free energy profiles are sufficiently low and the histograms of the RC1 reaction-coordinate for 121 umbrella windows show reasonably significant mutual overlaps, indicating statistical convergence of the result (Fig. S7†). Furthermore, we computed the free energy profiles for the am_6_αCD translocation at pH 8 obtained from 10 ns and 20 ns simulation times for the 121 umbrella windows and the free energy profiles show good convergence (Fig. S7†). The simulation protocol allowed a relative comparison of free energy profiles and barrier heights in native and truncated CymA across various pH conditions and cyclodextrins (CD). We describe the CD translocation event as a consequence of two contrasting phenomena: free energy barrier height and free-energetic stabilization of the CD inside the pore. We show snapshots of the CD translocation events through the pore at pH 8.0, referring to different locations on the free energy profile (Fig. 3a and b). We show the polar interactions between am_6_αCD and acidic amino acid residues (GLU and ASP) in two configurations of the CD translocating through the native CymA at an RC1 value of approximately −0.5 nm, where the CD is residing very close to the center of the pore. The am_6_αCD had favorable polar interactions (hydrogen-bonding) with multiple negatively charged amino acid residues at pH 8.0 (E32, E115, E135, E220 and D3). The stronger polar interactions between the am_6_αCD and the larger number of negatively charged amino acid residues lead to facilitated translocation of am_6_αCD (Fig. 3c). The negative electrostatic potential distribution demonstrates that the pore surface is lined with negatively charged amino acid residues near the CD entry region at pH 8.0 (Fig. 3d). At this pH, strong electrostatic attractive interactions between negatively charged residues with the positively charged CD strongly stabilize the am_6_αCD upon entering the pore. However, these electrostatic and polar interactions are relatively weaker at pH 4.5 as many negatively charged amino acid residues are neutral (Fig. 3c). The resulting positive electrostatic potential distribution inside the pore results in weaker interaction with am_6_αCD. Since favorable binding of CD to the protein is essential for the successful translocation of the CD to the periplasmic side, the translocation is more effectively facilitated at pH 8.0 than at pH 4.5, which agrees with single-channel recordings (Fig. 3).

### Interaction of cationic cyclodextrin with truncated CymA at different pHs

**Fig. 4:**
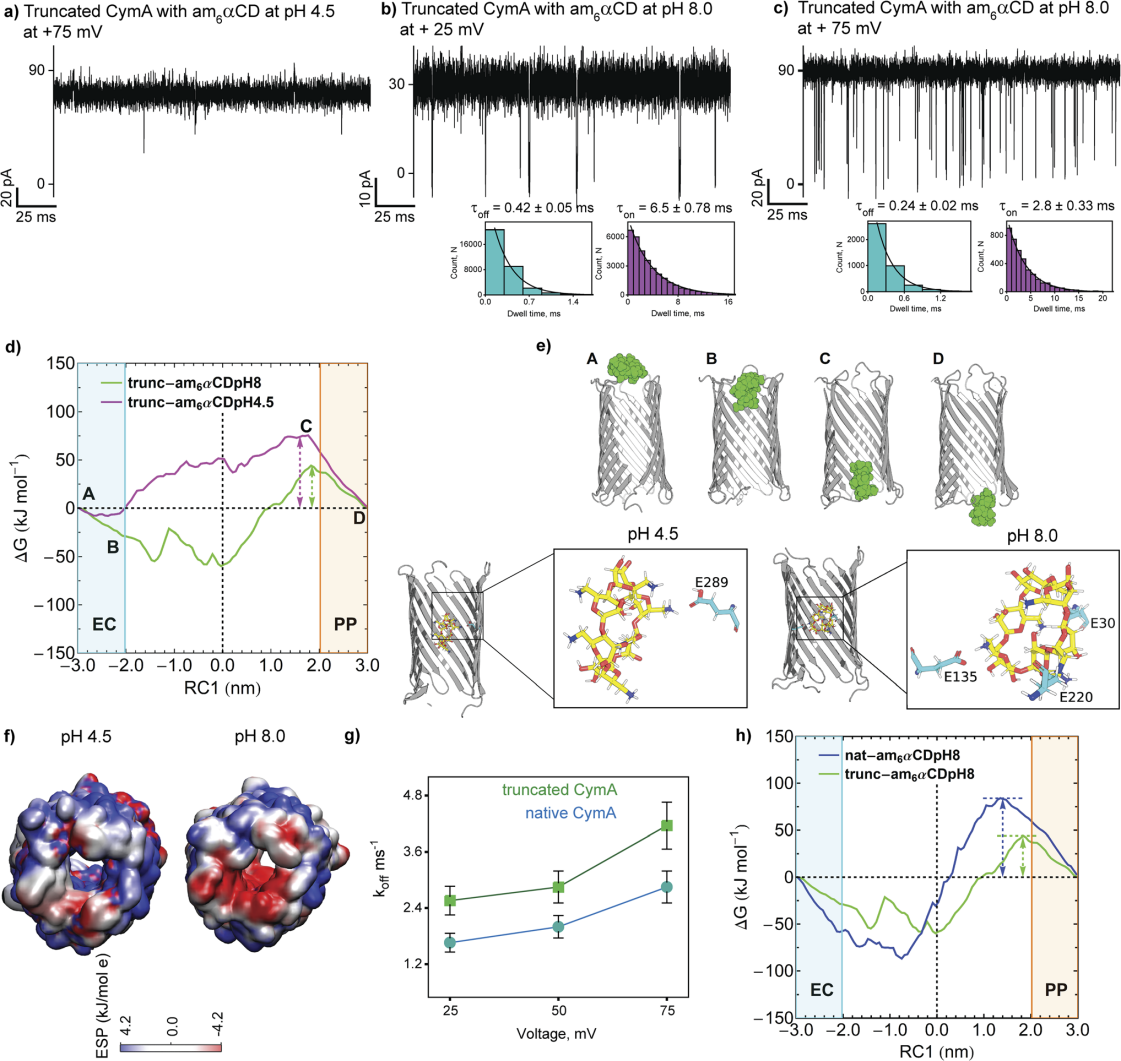
Interaction of am_6_αCD with truncated CymA. (a) Electrical recordings of single truncated CymA in the presence of am_6_αCD (10 μM, *trans*), at +75 mV at pH 4.5 and (b) at +25 mV and (c) +75 mV at pH 8.0. Insets show the corresponding *τ*_off_ and *τ*_on_ dwell time histograms of CD blocking fitted with a monoexponential probability function. (d) One-dimensional free energy profile of am_6_αCD translocation through truncated CymA at pH 8.0 (green curve) and pH 4.5 (magenta curve). (e) Multiple locations of the free energy profile are labeled from A to D, referring to various stages of the CD translocation event. EC and PP refer to the extracellular and periplasmic regions, respectively. The protein is shown in cartoon (grey) representation and the CD is shown as space-filling spheres (green). Snapshots of am_6_αCD translocation at pH 4.5 and 8.0 showing the acidic amino acid residues involved in hydrogen bonding interactions with CD. The CD and amino acid residues are shown in stick representation. (f) Electrostatic potential distribution (kJ mol^−1^ e) on the truncated protein surface at pH 4.5 and 8.0. (g) A plot showing *k*_off_ as a function of voltage indicating am_6_αCD translocation comparison between native and truncated CymA at pH 8.0. Mean values (±s.d.) from three different experiments are shown. (h) One-dimensional free energy profile of am_6_αCD translocation through the truncated (green) and native (blue) CymA at pH 8.0. Electrolyte: 1 M KCl, 10 mM citrate, pH 4.5 and 1 M KCl, 10 mM HEPES, pH 8.0. The current signals were digitally filtered at 7 kHz.

Next, we examined the interaction of am_6_αCD with truncated CymA under different pH conditions to establish the role of the N terminus in regulating molecular transport (Fig. 4 and Table S3†). The addition of 10 μM am_6_αCD to the *trans* side of the pore at pH 4.5 did not produce any ion current blockages, indicating low binding affinity of the CD with the pore (Fig. 4a and S8†).

However, adding 10 μM am_6_αCD to the *trans* side of the pore at pH 8.0 produced pronounced ion current blockages at positive voltages, indicating high binding affinity of the CDs to the pore (Fig. 4b and S8†). The *k*_on_ and *k*_off_ increased with the voltage consistent with the translocation of the CDs across the pores (Fig. 4b and c and Table S3†). To elucidate the impact of the N terminus on the free energetics of the CD translocation, we further calculated 1D-free energy profiles as a function of RC1, obtained from umbrella sampling simulations of am_6_αCD translocation through truncated CymA (Fig. 4d) at pH 4.5 and 8.0. We obtained a free energy barrier of approximately 75 kJ mol^−1^ for CD translocation through truncated CymA at pH 4.5 compared to a smaller barrier of 42 kJ mol^−1^ at pH 8.0 (Fig. 4d). The am_6_αCD becomes much more stabilized upon entering the pore at pH 8.0 than at pH 4.5, owing to the favorable intermolecular interactions between the negatively charged amino acid residues at pH 8.0 (Fig. 4d and e). Notably, CD translocation through the truncated CymA at pH 8.0 is much more favorable due to the facilitated binding of the CD inside the pore (Fig. 4d and e). Thus, the negative electrostatic potential provides a major origin of facilitated CD translocation (Fig. 4f). The reduced stabilization of the CD at pH 4.5 inside the truncated pore can be attributed to muted electrostatic interactions with neutral and acidic amino acid residues, resulting in poor or no translocation.

Interestingly, am_6_αCD binds to native and truncated CymA at pH 8.0 with distinct binding kinetics (Fig. 4g). The am_6_αCD blocked native CymA with the average dwell time of the blockage (*τ*_off_) of 0.55 ± 0.06 ms, whereas it blocked truncated CymA with *τ*_off_ of 0.32 ± 0.04 ms at +50 mV. Notably, *k*_off_ through truncated CymA was higher than native CymA, indicating faster translocation of am_6_αCD through the truncated pore and subsequently demonstrating the presence of the N terminus inside native CymA during molecular transport (Fig. 4g). To elucidate the role of N terminus truncation on the CD translocation, we further compared the free energy profiles for am_6_αCD translocation through the native (blue curve) and truncated CymA (green curve) at pH 8.0 (Fig. 4h). The free energy barrier for am_6_αCD translocation through truncated CymA (∼42 kJ mol^−1^) is reduced by a factor of two compared to the barrier for translocation through native CymA (∼80 kJ mol^−1^). We compared the electrostatic potential surfaces of the truncated CymA with that of native CymA at pH 8.0 and an open space near the periplasmic region can be observed for the truncated pore (Fig. 3d and 4f). The space refers to the absence of the N terminus after truncation of native protein conformation. We posit that the N terminus provides a steric barrier for the translocation of am_6_αCD across the native CymA and the truncation of the segment eventually makes it much easier for the am_6_αCD to translocate through the pore (Fig. 4g and h). However, removing the N terminus does not alter the pore's affinity site for electrostatic am_6_αCD binding and translocation.

### Modulation of the pore's affinity site for substrate translocation and charge reversal

**Fig. 5:**
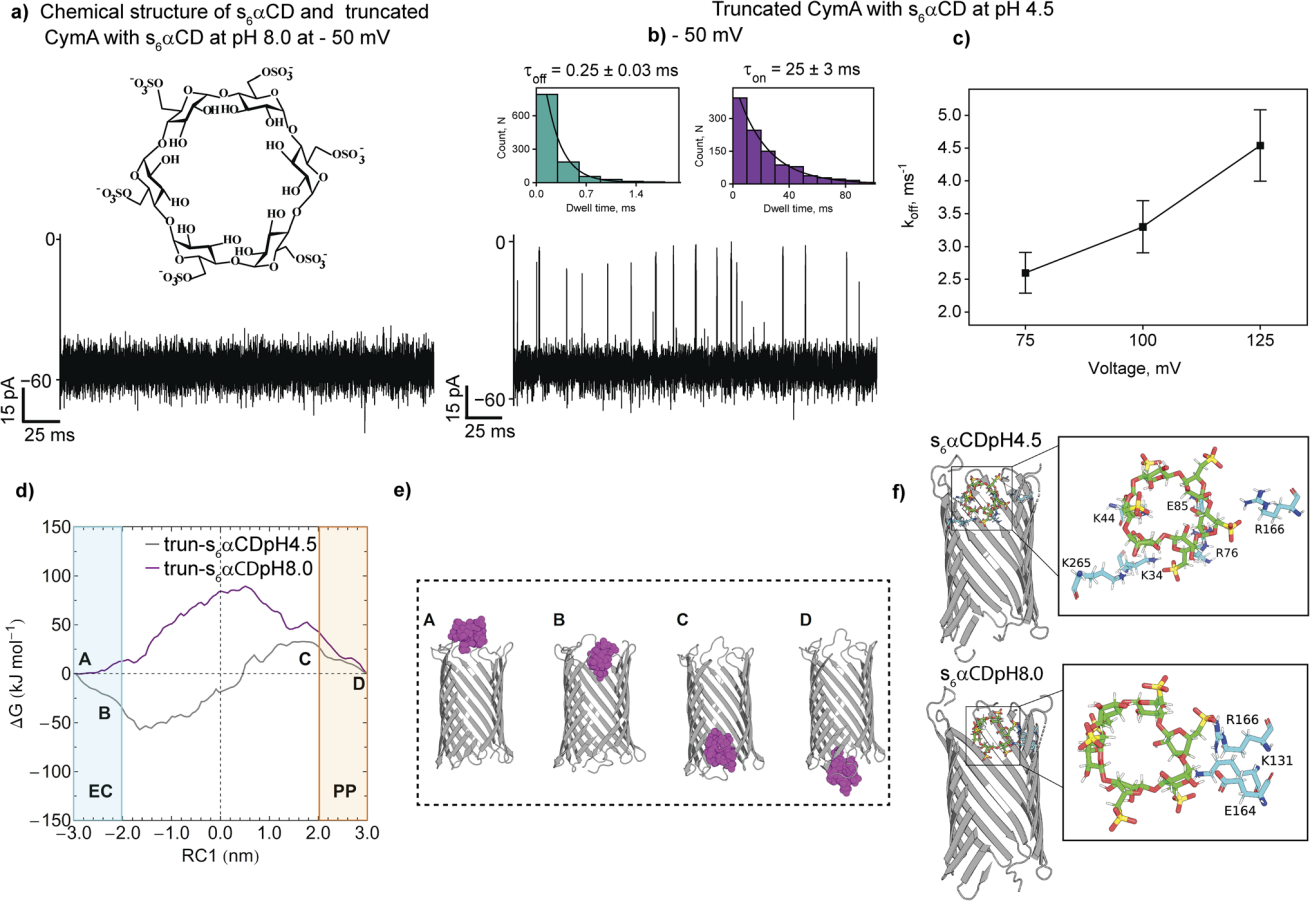
Interaction of s_6_αCD with truncated CymA. (a) Electrical recordings of single truncated CymA in the presence of s_6_αCD (100 μM, *trans*) at −50 mV at pH 8.0 and (b) −50 mV at pH 4.5. Electrolyte: 1 M KCl, 10 mM citrate, pH 4.5 and 1 M KCl, 10 mM HEPES, pH 8.0. (c) A plot of *k*_off_*versus* voltage at pH 4.5 is shown. Mean values (±s.d.) from three different experiments are shown. The current signals were digitally filtered at 7 kHz. (d) One-dimensional free energy profile of s_6_αCD translocation through truncated CymA at pH 8.0 (magenta) and 4.5 (grey) as a function of RC1. (e) Multiple locations on the free energy profile are labeled from A to D, referring to various stages of the CD translocation. The s_6_αCD is shown as purple space-filling spheres. (f) Snapshots of s_6_αCD translocation at pH 4.5 and 8.0 showing the charged amino acid residues involved in hydrogen bonding interactions with CD. The CD and amino acid residues are shown in stick representation.

Next, we investigated the interaction of an anionic hexasaccharide, s_6_αCD, with the CymA pores under different pH conditions to gain insights on the charge-selective translocation of CDs (Fig. 5, S9 and S10†). Adding 10 μM s_6_αCD to the *trans* side of native CymA did not produce ion current blockages at different voltages at pH 8.0 (Fig. S9†). We further increased the concentration of s_6_αCD to 100 μM, resulting in very short, less frequent ion current blockages indicating negligible interaction of the CDs with the pore surface (Fig. S9†). A weak binding pattern of s_6_αCD with truncated CymA was observed at pH 8.0 (Fig. 5a and S10†).

Next, we studied the interaction of s_6_αCD with truncated CymA at pH 4.5. The addition of s_6_αCD to the *trans* side of the pore produced well-defined frequent ion current blockages at negative voltages starting from −50 mV as CDs were electrophoretically driven into the pore (Fig. 5b and c and S10†). No blockages were observed at positive voltages, confirming the electrostatic binding of the CDs with the pore surface (Fig. S10†). The *k*_on_ and *k*_off_ increased with an increase in the voltage from −75 mV to −125 mV, indicating successful translocation of s_6_αCD at pH 4.5 (Fig. 5c and S10 and Table S4†). To study the s_6_αCD translocation along the truncated pore in atomistic detail, we calculated a one-dimensional free energy profile as a function of the reaction coordinate RC1 at pH 4.5 (grey) and 8.0 (magenta) (Fig. 5d). The free energy profile and the corresponding snapshots showing various stages of the CD translocation revealed the destabilization of CDs inside the pore at pH 8.0 upon entering the pore from the extracellular side (Fig. 5e). The instability of s_6_αCD at pH 8.0 results from the destabilizing electrostatic repulsion between anionic CD and negatively charged residues lined along the pore (Fig. 5e and f). In contrast, at pH 4.5, the s_6_αCD becomes highly stabilized inside the pore and strong positive electrostatic potential distribution is dominated by the positively charged amino acid residues (Lys, Arg and His) (Fig. 5f). Thus, s_6_αCD engaged in more favorable electrostatic binding interactions with the positively charged residues, resulting in effective CD translocation at pH 4.5 in agreement with experimental results (Fig. 5a–c and f). Importantly, we show the modulation of the charge-based affinity site of the pore by altering the solutions' pH for the effective transport of charged CDs without protein engineering. For example, under basic conditions (pH 8.0), the acidic residues are deprotonated, enhancing the cation selectivity and promoting strong binding of am_6_αCD. In contrast, at pH 4.5, the acidic residues lining the pore lumen are protonated, reducing the cation-selectivity and establishing charge-selective translocation of anionic s_6_αCD. Additionally, we examined the interaction of neutral alpha cyclodextrin (αCD) with truncated CymA to confirm the pH effect on the substrate binding. At pH 4.5 and 8, αCD binds to the pore with low binding affinity in agreement with the charge selectivity of the pore. As expected, αCD produced numerous strong ion current blockages through the truncated CymA at pH 6.0, indicating a high affinity of the CD with the pore surface (Fig. S11†). We further studied the αCD binding with native and truncated CymA and correlated the binding affinity with the pore at pH 6.0. Neutral αCD binds to the native and truncated CymA, producing distinct ion current blockages. For example, αCD blocked native CymA with *τ*_off_ 0.620 ± 0.05 ms and truncated CymA with *τ*_off_ 0.4500 ± 0.04 ms at +50 mV (Fig. S11†). This data indicates the faster translocation of neutral αCD through the truncated CymA, most likely due to the absence of the N terminus segment establishing its functional relevance in the substrate translocation. Accordingly, we emphasize that CymA can be used as a versatile nanopore sensor for single-molecule sensing of cationic, anionic and neutral alpha cyclodextrins under different experimental pH conditions (Fig. S12†).^33,36–43^ Next, we confirmed this charge reversal of pores with pH using a miniaturized bilayer workstation called orbit16 that allows simultaneous recordings of multiple highly stable artificial lipid bilayers horizontally.^44^ Am_6_αCD blocked the truncated CymA pores at pH 8.0, indicating high binding affinity, whereas anionic s_6_αCD did not produce any ion current blockages at pH 8.0 (Fig. S13†). Here, we show that the pH-controlled charge reversal exhibited by CymA can be exploited for single-molecule sensing of differently charged biomolecules without protein engineering, demonstrating the functional versatility of the pore. Notably, the effect of pH on the charge of CDs within the studied range is negligible.

### Conformational dynamics of the N terminus segment during the substrate transport

**Fig. 6:**
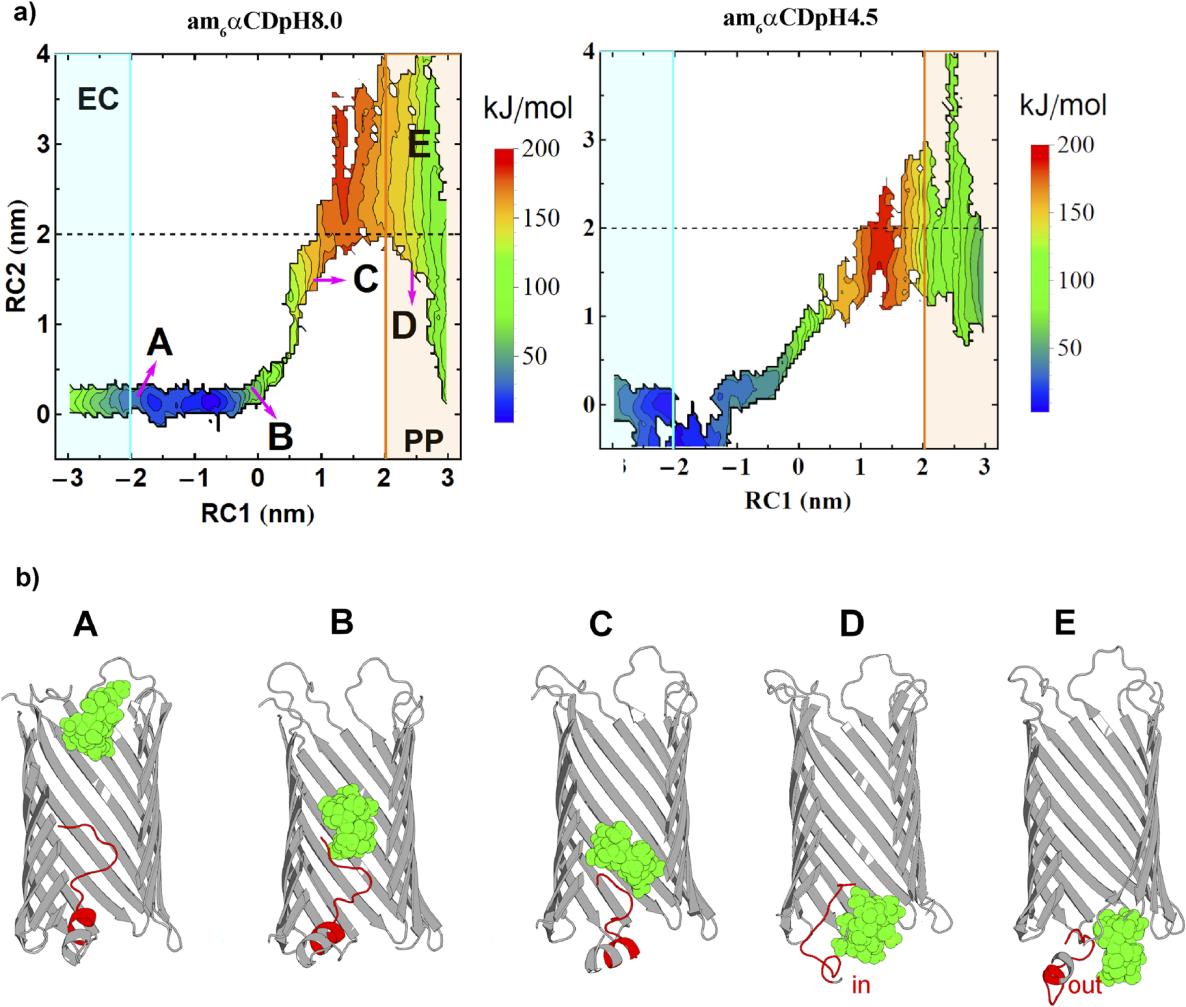
Dynamics of the N terminus during the cyclic sugar transport across CymA. (a) 2D-FESs obtained for am_6_αCD translocation at pH 8.0 and 4.5 are shown. The black dashed line refers to a protein–NtermCA distance of ∼2.0 nm. The protein–NtermCA distances of more or less than ∼2.0 nm can be designated if the loop is inside or outside the pore lumen. (b) Multiple snapshots of the N terminus segment dynamics relative to various stages of CD translocation are shown for various regions of the 2D-FES at pH 8.0. Snapshots A and B refer to the N terminus region residing close to the pore center when the CD is close to the entrance and center of the pore. Snapshot C refers to the CD crossing the centre of the pore. Snapshots D and E show the possible dynamics of the N terminus during the CD translocation across the pore. The am_6_αCD is shown in the green space-filling sphere representation, the protein in the gray cartoon representation, and the N terminus in red.

To understand the dynamics of the N terminus, we calculated two-dimensional free energy surfaces (2D-FESs) as a function of the distance vectors between the protein and the CD COMs (RC1) and the protein COM and the N terminus residue-CA (RC2, CA refers to an alpha carbon atom) atom, along the *z*-direction. The 2D-FESs were obtained for am_6_αCD translocation by calculating the joint probability distributions of RC1 and RC2 from the umbrella sampling simulations (Fig. 6a).

The dashed black line at an RC2 value of ∼2.0 nm can be rendered as the borderline region to locate whether the N terminus is inside or outside the pore lumen (Fig. 6). A protein–NtermCA distance (RC2) value of 0 nm refers to the N terminus residing completely inside the pore lumen with the residues present approximately at the center of the pore (Fig. 6). Any value above zero indicates the outward motion of the N terminus towards the periplasmic region. The CD translocation through the pore from the extracellular to the periplasmic region is described by the gradual increase in the pore–CD COM distance (RC1) from −3.0 nm to +3.0 nm. Thus, the 2D-FES can provide a qualitative description of the CD translocation event as a function of the position of the N terminus relative to the pore center of mass. The FES for pH 8.0 and 4.5 is qualitatively similar for RC1, ranging from ∼−3 to 0.0 nm. In this region, the RC2 value fluctuates from 0 to 0.2 nm, which indicates that the CD movement does not affect the N terminus dynamics when the CD is far from the center of the protein and does not cross protein COM (Fig. 6a). Various basins in the free energy surface have very high values (red-colored regions). These basin regions refer to the locations of the phase space that are probabilistically unlikely for the CD to explore, which the current enhanced sampling approach enabled us to sample exhaustively. With an increasing value of RC1 (>0 nm), *i.e.*, with further movement of the CD towards the periplasmic region, the RC2 value is altered significantly. As soon as the CD reached the center of the pore, the CD movement started to perturb the motion of the N terminus region. The dashed line in 2D-FESs refers to the location of the N terminus inside or outside the pore lumen (Fig. 6a). The RC2 value greater than or less than 2.0 nm indicates the N terminus residing outside or inside the pore lumen, respectively. The 2D-FESs at both pHs demonstrate that the N terminus can undergo a combination of “in” and “out” motions during the translocation (Fig. 6a). To elucidate the correlation between the N terminus dynamics and CD translocation, we have shown snapshots of the am_6_αCD translocating across the native CymA at pH 8.0, referring to different basins of the 2D-FES (Fig. 6b). Snapshots A, B, C, D and E show the position of the flexible N terminus at various stages of the CD translocation. Snapshot A refers to the N terminus residing very close to the center of the protein during CD translocation from the extracellular region. Snapshot B refers to the N terminus residing close to the protein COM when the CD is spatially very close to the protein COM. Snapshot C refers to a situation where the motion of the N terminus is highly perturbed by the CD movement (RC2 ∼1.5 nm) where the CD molecule is translocated past the COM of the pore (RC1 ∼0.93 nm). Interestingly, the N terminus shows significant probabilities of both “in” and “out” motions during CD translocation across the pore. Snapshots D (RC2 ∼1.55 nm) and E (RC2 ∼3 nm) refer to the “in” and “out” motions of the N terminus when the CD has translocated towards the periplasmic region of the pore, respectively (Fig. 6b and S14†). To obtain a residue-specific description of the ligand translocation near the periplasmic region, we show the amino acid residues involved in polar intermolecular interactions with the ligand at pH 8 (Fig. S15†). Furthermore, we calculated a minimum free energy path (MFEP) based on a post-string method described by Morita *et al.*^45^ We show the MFEP and steered MD simulation trajectory path projected onto the 2D-FES as a function of RC1 and RC2 (Fig. S16†). The MFEP and steered MD trajectory path show good agreement. Here, we define a new translocation paradigm where the N terminus exhibits a dynamic equilibrium of “in” and “out” motions during am_6_αCD translocation across the pore (Fig. 6). Importantly, the 2D-FES plots validate a critical experimental hypothesis that the N terminus and cyclic sugar co-exist in the pore during translocation (Fig. 6b, snapshot D and S14†). Most precedent MD simulations have explored substrate (cyclic sugars) translocation through a truncated CymA (residues 1–15 removed).^17,21,46^ In this study, we show a highly original atomistic molecular dynamics investigation of substrate translocation through the native CymA that provides insights into a new translocation paradigm, which will serve as a new benchmark for transporter studies. Using free energy simulations, we show how translocation through a protein pore depends on the strength of the substrate–channel interaction, specifically on a local affinity site.^3,35^ Importantly, experimentally deriving a translocation energetic profile and estimating the associated barrier in a native condition (*i.e.*, a state devoid of external bias) is currently not feasible. In this regard, devising free energy-based computer simulations that can realize such a scenario provides much required insights and proof-of-concept of experimentally posited pH effects, besides rendering a high-resolution molecular view of the overall translocation process.^3,35^

To date, the activation of the dynamic segments in controlling molecular transport is reported mainly with ligand-gated active transporters.^1,4,24^ Our studies shed light on the conformational dynamics of the N terminus segment in the outer membrane porin, CymA, and its influence in modulating the passive transport of cyclic sugars.^17,22^ Unlike active transporters, we propose that the CymA cannot be classified as a ligand-gated channel as the substrate binding did not expel the N terminus entirely from the pore.^4,17,24^ We examined the pore properties of fully truncated CymA (ΔN21), where the N terminus is entirely removed in single-channel electrical recordings. The pore fluctuated between different sub-conductance states, indicating multiple pore conformations, suggesting the importance of the N terminus for pore stability (Fig. S17†). Most previous studies have targeted antibiotic permeation across general diffusion porins and recognized the impact of a charged constriction zone and small loops in regulating transport.^2,3,7,11,35^ Similarly, we suggest the antibiotics translocation across CymA due to the presence of charged residues lining the pore lumen that acts as a binding site promoting molecular transport. Accordingly, we characterized the interaction of antibiotics with CymA in single-channel recordings. Adding aminoglycoside antibiotic neomycin (10 μM) to the *trans* side of the truncated CymA at low salt conditions (0.15 M KCl) results in the well-defined ion current blockages at +25 mV and +50 mV (Fig. S18†). This indicated the binding affinity of the neomycin to the pore surface, facilitating voltage-dependent translocation of antibiotics. Therefore, we emphasize that CymA can be exploited as a transporter for hijacking antibiotics into the bacterial cell as orthologs of CymA are identified in priority bacterial pathogens such as *Klebsiella pneumoniae*.^17^ In the future, translocation kinetics of antibiotics and substrates will be quantified across the pores by mimicking *in vivo* conditions, such as assembling asymmetric outer membranes made up of lipopolysaccharides and natural bacterial membrane phospholipids.^14,47^ The current framework of enhanced sampling nature of computer simulation allows us to provide a free energy perspective of the substrate transport across the CymA porin. In the future, it would be potentially interesting to explore the kinetic aspect of the process *via* a combination of adaptively explored simulation trajectory and Markov state models.^48^ Further, we will measure the antibiotic binding specifically at the Donnan potentials (0 to 30 mV) in low salt buffers close to natural outer membrane conditions. Alternatively, cyclic sugars, peptides, and cucurbiturils can be targeted as CymA blockers against bacterial pathogens.^49–51^

## Conclusion

In this study, we present a bacterial transporter, CymA, with a unique structural conformation, which passively transports cyclic sugars (∼900 Da). We decipher the interplay between CymA's structural components and translocation functionality through an integrated approach encompassing experimental electrical recordings and molecular dynamics simulations. Importantly, we highlight the influence of dense charge distribution in the pore for efficient binding and translocation of substrates. We unveil pH-responsive substrate binding site modulation within the CymA, offering an unprecedented glimpse into pH-driven control of substrate transport. Further, we show that the N terminus is highly flexible during substrate transport and exhibits a conformational dynamic mechanism regulating large molecular transport.

## Experimental section

### Single-channel electrical recordings

Electrical recordings were performed using planar lipid bilayers formed of 1,2-diphytanoyl-*sn*-glycero-3-phosphocholine (DPhPC, Avanti Polar Lipids).^52^ Bilayers were formed across a polytetrafluoroethylene (Teflon) film aperture (25 μm thick, ∼100 μm in diameter) (Goodfellow, Cambridge), which divided the bilayer chamber into *cis* and *trans* compartments (600 μL each). The aperture was prepainted with hexadecane in *n*-pentane (2 μL, 5 mg mL^−1^) on each side and *cis* and *trans* compartments were filled with the electrolyte (1 M KCl, 10 mM HEPES, pH 8.0 and citrate buffer for pH 4.5) and DPhPC in *n*-pentane (2.5 μL, 10 mg mL^−1^). The monolayer was made by lowering the electrolyte level below the aperture in the film and raising it again to produce a bilayer at the aperture. The purification of the proteins was carried out as described previously.^17^ Purified proteins were added to the *cis* side for insertion at the applied voltage of +200 mV. The *cis* compartment was attached to the grounded electrode, while the *trans* compartment was connected to the live electrode attached to the amplifier. A potential difference was applied to the system through a pair of silver–silver chloride electrodes. The current was amplified using an Axopatch 200B amplifier, digitized with a Digidata 1550B and recorded with the pClamp 10.6 acquisition software (Molecular Devices, CA) with a low-pass filter frequency of 10 kHz and a sampling frequency of 50 kHz. Few current signals were digitally filtered at 7 kHz and 2 kHz. The mean dwell time of CD blocking (*τ*_off_) and time between successive blocking (*τ*_on_) were calculated in a single-channel analysis and the dissociation rate (*k*_off_ = 1/*τ*_off_) and the association rate (*k*_on_ = 1/*τ*_on_ × *C*) were determined.

### Molecular dynamics simulations

CymA, lipid and ions were modelled using the CHARMM36 all-atom force field, while the water molecules were modelled using TIP3P-CHARMM parameters.^53,54^ Both cationic and anionic forms of CD were used to simulate their translocation process across native and truncated CymA. The cationic and anionic αCDs are am_6_αCD and s_6_αCD, having total charges of +6 and −6, respectively. All systems and their respective size have been detailed in Tables S5 and S6.† We performed a series of umbrella sampling simulations to study the energetics of the CD translocation process (ESI text†). The CHARMM general force field (CGENFF) CD-ligand parameters were obtained using the ligand reader and modeler plugin in the CHARMM-GUI web server.^55,56^ The distance vector between the protein's COMs (centers of mass) and the CD along the *z*-axis was selected as the preferred reaction coordinate (RC1) to describe the CD translocation event. Initial configurations for umbrella sampling simulations were obtained from a non-equilibrium-steered MD simulation that forced the CD to translocate from the extracellular side of the protein to the periplasmic side through the protein core. For each umbrella sampling simulation, we have run 121 umbrella sampling windows for reaction-coordinate values ranging from −3.00 nm to +3.00 nm. Each umbrella sampling window was simulated for 10 ns. All simulations were performed using the GROMACS-2018 software package.^57^ The free energy profile as a function of the reaction coordinate (RC1) was obtained by reweighting the probability distributions of the reaction coordinate using the WHAM (Weighted Histogram Analysis Method) tool implemented in the GROMACS MD simulation software package.^58^

## Data availability

All data are available in the article, in the ESI† and from the authors upon request.

## Author contributions

DV performed single-channel current recordings. BBM performed molecular dynamics simulations. DV and SK purified the proteins. DV, KRM, CW, NF and MW analyzed the current recordings. DV, BM, JM and KRM conceived the study and designed the experiments. JM and KRM supervised the study. All authors wrote and approved the paper.

## Conflicts of interest

There are no conflicts to declare.

## Supplementary Material

SC-015-D4SC00345D-s001
