# Supplementary material for: Conformational flexibility driving charge-selective substrate translocation across a bacterial transporter
Source: Chem Sci. 2024 May 13;15(24):9333–44. doi: 10.1039/d4sc00345d (PMC11186346; doi:10.1039/d4sc00345d)
Supplement: SC-015-D4SC00345D-s001 [file SC-015-D4SC00345D-s001.pdf]

Electronic Supplementary Material (ESI) for Chemical Science.
This journal is © The Royal Society of Chemistry 2024

Supporting Information

Conformational Flexibility Driving Charge-Selective Substrate Translocation Across Bacterial Transporter

Devika Vikraman[a,b†], Bibhab Bandhu Majumdar[c†], Sharavanakkumar SK[a], Conrad Weichbrodt[d], Niels Fertig[d], Mathias Winterhalter[e,f], Jagannath Mondal*[g], Kozhinjampara R Mahendran*[a]

[†]Contributed equally to this work

[a]Transdisciplinary Research Program, Rajiv Gandhi Centre for Biotechnology, Thiruvananthapuram 695014, India.

[b]Manipal Academy of Higher Education, Manipal, Karnataka, India-576104

[c]School of Advanced Sciences, VIT-AP University, Amaravati, Andhra Pradesh 522237, India

[d]Nanion Technologies GmbH, Munich, 80339 Germany.

[e]School of Science, Constructor University, Campus Ring 1, 28759 Bremen, Germany

[f] Center for Hybrid Nanostructures (CHyN), Universität Hamburg, Luruper Chaussee 149, Hamburg 22761 Germany

[g]Tata Institute of Fundamental Research, Hyderabad, Telangana, India-500046

*To whom correspondence should be addressed

e-mail: mahendran@rgcb.res.in

jmondal@tifrh.res.in

Contents

Supplementary Materials 3

Supplementary Text 3

Supplementary Table 6

Table S1: The pKa values were obtained from titratable amino acid residues in CymA pores using the PropKa program. 6

Table S2: $Am_6\alpha CD$-binding kinetics with native CymA at pH 8.0. 10

Table S3: $Am_6\alpha CD$-binding kinetics with truncated CymA at pH 8.0. 10

Table S4: $S_6\alpha CD$-binding kinetics with truncated CymA at pH 4.5. 11

Table S5: Description of the different components of the simulation systems. 11

Table S6: Description of simulation systems. 12

Supplementary Figures

Fig. S1: CymA structures, orientation and electrophoretic pulling of cationic analytes. 13

Fig. S2: Gating pattern of native CymA. 14

Fig. S3: Gating pattern of truncated CymA. 15

Fig. S4: Interaction of $am_6\alpha CD$ with native CymA under different pH conditions. 16

Fig. S5: Representative configuration of the hydrated CymA embedded in DPhPC lipid bilayer MD-simulation system with $am_6\alpha CD$. 17

Fig. S6: Multiple Configurations of the $am_6\alpha CD$ while translocating through the native CymA. 18

Fig. S7: A one-dimensional free energy profile was obtained for $am_6\alpha CD$. translocation through native CymA at pH 4.5 and 8. 19

Fig. S8: Interaction of $am_6\alpha CD$ with truncated CymA at pH 4.5 and 8. 20

Fig. S9: Interaction of $s_6\alpha CD$ with native CymA under different pH conditions. 21

Fig. S10: Interaction of $s_6\alpha CD$ with truncated CymA under different pH conditions. 22

Fig. S11: Interaction of neutral αCD with CymA pores at different pHs. 23

Fig. S12: Interaction of $am_6\alpha CD$, $s_6\alpha CD$ and αCD with truncated CymA. 24

Fig. S13: Interaction of $am_6\alpha CD$ and $s_6\alpha CD$ with truncated CymA in orbit16. 25

Fig. S14: Molecular model of substrate translocation through CymA. 26

Fig. S15 CymA protein residues involved in polar interactions with $am_6\alpha CD$. 27

Fig. S16 A minimum free energy path (MFEP) obtained using a post-string approach. 28

Fig. S17: Single-channel properties of fully truncated CymA. 29

Fig. S18: Interaction of neomycin with truncated CymA. 30

References 31

Materials used:

The following materials were used for the study: 1,2-diphytanoyl-*sn*-glycero-3-phosphocholine (DPhPC, Avanti Polar Lipids), pentane (Sigma-Aldrich Merck), hexadecane (Sigma-Aldrich Merck), n-Octylpolyoxyethylene (Octyl-POE, Sigma-Aldrich Merck), hexakis-(6-amino-6-deoxy)-α-cyclodextrin hexahydrochloride ($am_6αCD$, AraChem Cyclodextrin-Shop), cyclic hexasaccharide sulfate ($s_6αCD$, AraChem Cyclodextrin-Shop), potassium chloride (Sigma-Aldrich Merck), 4-(2-hydroxyethyl)-1-piperazineethanesulfonic acid (HEPES, Sigma-Aldrich Merck), citric acid monohydrate (Sigma-Aldrich), Potassium phosphate dibasic and Dibasic potassium phosphate (Sigma-Aldrich Merck), 2-Propanol (Sigma-Aldrich Merck), all other reagents (Sigma-Aldrich Merck).

Supporting Text:

Molecular Dynamics Simulations

The missing residues in native CymA protein (residues 10 to 21) were modelled in CHARMM-GUI as described in the Methods section in the main text [1]. The protein was then protonated at pH 4.5 and 8 using the PDB2PQR webserver that uses PropKa to calculate the pK_a values of the titratable amino acid residues (see Table S1) [2, 3]. The charge of the native CymA protein at pH 4.5 and 8.0 was determined to be +14 and -6, respectively. The CymA protein was inserted in a DPhPC lipid bilayer containing 115 lipid molecules, each on the upper and the lower leaflets, resulting in 230 lipid molecules. The membrane-protein system was built in a tetragonal simulation box with box vectors: 10.2, 10.2 and 10.9 nm in the x, y and z-directions, respectively. The simulation system was solvated using TIP3P-charmm water molecules and neutralized by adding K^+ and Cl^- ions to obtain 0.15 M KCl concentration. The simulation system with CymA, DPhPC lipid bilayer (PHPC in CHARMM-GUI), water and ions was prepared using the Membrane-Builder utility in the CHARMM-GUI webserver[1]. A three-dimensional structure of the neutral α-cyclodextrin was obtained from the PubChem database (PubChem id: 444913). The six hydroxymethyl groups ($-CH_2OH$) in neutral- α-cyclodextrin (αCD) were modified to methylammonium cations ($-CH_2-NH_3^+$) to obtain a cationic-α-cyclodextrin ($am_6αCD$) molecule with +6 charge using the PYMOL software package. The geometry-optimized structure of the αCD molecule was then used as a cationic α-cyclodextrin. The geometry optimization was performed using the Avogadro software package. The hydroxymethyl groups in neutral- α-cyclodextrin were modified to methyl sulfonate anions ($-CH_2-SO_3^-$) for obtaining an anionic α-cyclodextrin ($s_6αCD$) molecule with -6 charge. To

prepare the truncated CymA, we removed the first 15 residues of the native CymA and followed a similar protocol as described for native CymA for protonating ionizable amino acid residues at pH 4.5 and 8.0. The protein, lipids and ions were parameterized using CHARMM36 forcefield while the CD-based ligands were modelled using CHARMM general force field (CGENFF forcefield) [4-7]. Tables S5 and S6 provide the details for each system simulated in the present investigation.

The CD was inserted in the membrane protein system towards the extracellular side aligned with the z-axis. After an initial energy minimization using the steepest descents algorithm, the membrane-protein system was then equilibrated for 10 ns using a canonical (NVT) ensemble, followed by 20 ns equilibration using an isothermal and isobaric ensemble (NPT) with position restraints on protein, lipid and ligand heavy atoms. The Verlet cutoff scheme was employed with a minimum cutoff of 1.2 nm for the Lenard Jones interaction and short-range electrostatic interactions throughout the simulation[8]. Long-range electrostatic interactions were treated by the Particle Mesh Ewald (PME) summation method [9]. All bonds connected to hydrogen atoms were constrained using the LINCS algorithm [10]. The bonds and the angles of TIP3P water molecules were constrained using the SETTLE algorithm [11]. A time-step of 2 fs was used for all MD simulations.

During equilibration, the temperature and pressure were kept constant at 303.15 K and 1 bar using the Berendsen thermostat and barostat with time constants of 1 ps and 5 ps, respectively [12]. However, the Nose Hoover thermostat and Parinello-Rahman barostat were used for all production runs [13, 14]. The equilibrated membrane-protein system was then subjected to steered MD simulation, where the CD was steered through the CymA from the extracellular to the periplasmic region by pulling the CD very slowly across the pore. The cyclodextrin was pulled through the pore slowly along the z-direction using a pull force with a force constant of 100 kJ mol^{-1} nm^{-2} and a pulling rate of 0.1 Å/ns. The steered MD simulations were run sufficiently long for the CD to translocate and move out of the pore. The snapshots from the steered MD simulations were used as initial configurations for the umbrella sampling simulations. We have run 121 umbrellas for reaction-coordinate values ranging from -3.00 nm to +3.00. The negative and positive values of the reaction coordinate refer to the CD translocation from the extracellular to the periplasmic side. Each umbrella was run for 10 ns total simulation time. The harmonic restraint force constant was set to 1000 kJ mol^{-1} nm^{-2} for umbrella sampling simulations, ensuring good overlap between adjacent umbrella histograms. The free energy

profile as a function of the reaction coordinate was obtained by reweighting the probability distributions of the reaction coordinate using the WHAM (Weighted Histogram Analysis Method) tool implemented in the GROMACS MD simulation software package[15]. All MD simulations were run using the GROMACS-2018 software simulation package. Periodic boundary conditions were employed in x, y, and z-directions. All snapshots were obtained using the molecular visualization packages PyMol and VMD[16].

The details of the simulation systems are shown in Table S5 and Table S6, respectively. The simulation systems involving translocating the $am_6\alpha CD$ at pH 8 and 4.5 are designated nat-$am_6\alpha CD$pH8 and nat-$am_6\alpha CD$4.5, respectively. The simulation system involving translocating $s_6\alpha CD$ at pH 8 and 4.5 across the truncated CymA is designated trunc-$s_6\alpha CD$pH8 and trunc-$s_6\alpha CD$pH4.5, respectively. The simulation systems with $am_6\alpha CD$ translocating through truncated CymA at pH 8 and 4.5 are referred to as trunc-$am_6\alpha CD$pH8 and trunc-$am_6\alpha CD$pH4.5, respectively.

Supplementary Tables:

Residue	pKa inside protein	Model pKa
ASP 3	4.08	3.80
ASP 11	4.87	3.80
ASP 15	6.35	3.80
ASP 33	3.41	3.80
ASP 43	3.88	3.80
ASP 88	3.71	3.80
ASP 101	3.32	3.80
ASP 125	4.09	3.80
ASP 148	3.96	3.80
ASP 165	3.11	3.80
ASP 204	3.94	3.80
ASP 209	3.64	3.80
ASP 217	4.30	3.80
ASP 232	3.37	3.80
ASP 233	3.92	3.80
ASP 252	3.32	3.80
ASP 266	2.90	3.80
GLU 10	3.65	4.50
GLU 16	5.08	4.50
GLU 30	5.11	4.50
GLU 32	5.69	4.50
GLU 46	3.98	4.50
GLU 52	3.37	4.50
GLU 74	4.24	4.50
GLU 77	4.61	4.50
GLU 80	4.85	4.50
GLU 85	3.58	4.50

GLU 87	2.23	4.50
GLU 113	3.87	4.50
GLU 115	5.64	4.50
GLU 135	6.53	4.50
GLU 158	2.62	4.50
GLU 164	5.55	4.50
GLU 174	2.77	4.50
GLU 191	6.93	4.50
GLU 195	4.67	4.50
GLU 205	3.54	4.50
GLU 214	4.12	4.50
GLU 220	4.99	4.50
GLU 224	3.15	4.50
GLU 247	3.56	4.50
GLU 275	4.96	4.50
GLU 280	4.19	4.50
GLU 287	3.21	4.50
GLU 289	3.93	4.50
GLU 293	4.73	4.50
GLU 295	5.57	4.50
GLU 304	1.70	4.50
GLU 310	3.08	4.50
HIS 24	5.31	6.50
HIS 100	6.68	6.50
HIS 136	5.78	6.50
HIS 314	6.14	6.50
TYR 7	12.55	10.00
TYR 31	11.31	10.00
TYR 58	9.67	10.00
TYR 69	10.86	10.00

TYR 83	11.52	10.00
TYR 84	10.40	10.00
TYR 112	10.90	10.00
TYR 116	10.14	10.00
TYR 122	10.19	10.00
TYR 141	12.56	10.00
TYR 144	9.65	10.00
TYR 149	10.09	10.00
TYR 154	13.54	10.00
TYR 159	10.76	10.00
TYR 177	10.24	10.00
TYR 184	15.29	10.00
TYR 197	13.11	10.00
TYR 198	10.28	10.00
TYR 222	15.47	10.00
TYR 229	10.73	10.00
TYR 238	13.53	10.00
TYR 263	11.19	10.00
TYR 274	9.93	10.00
TYR 288	10.86	10.00
TYR 290	10.06	10.00
LYS 8	8.22	10.50
LYS 34	10.73	10.50
LYS 44	10.26	10.50
LYS 45	10.33	10.50
LYS 47	10.57	10.50
LYS 72	10.05	10.50
LYS 79	10.40	10.50
LYS 91	11.62	10.50
LYS 98	10.03	10.50

LYS 120	10.20	10.50
LYS 131	10.48	10.50
LYS 163	9.67	10.50
LYS 171	10.56	10.50
LYS 182	9.99	10.50
LYS 185	10.29	10.50
LYS 201	10.51	10.50
LYS 206	10.19	10.50
LYS 215	10.52	10.50
LYS 245	11.27	10.50
LYS 249	11.03	10.50
LYS 265	10.64	10.50
LYS 269	9.96	10.50
LYS 285	9.79	10.50
LYS 296	10.12	10.50
LYS 297	11.35	10.50
LYS 308	10.71	10.50
ARG 5	14.99	12.50
ARG 37	12.42	12.50
ARG 76	14.76	12.50
ARG 118	12.63	12.50
ARG 130	12.12	12.50
ARG 139	13.95	12.50
ARG 166	12.41	12.50
ARG 172	15.16	12.50
ARG 186	13.43	12.50
ARG 221	12.54	12.50
ARG 240	12.68	12.50
ARG 242	14.82	12.50
ARG 253	12.47	12.50

ARG 268	12.45	12.50
ARG 299	12.41	12.50
ARG 306	11.69	12.50

Table S1. The actual and model pKa values were obtained for titratable amino acid residues in CymA pores. The pKa values were obtained using the PDB2PQR web server that uses the PropK_a program. The estimated pKa values inside the protein, as predicted by the program, were employed to determine the charge of the protein at pH 4.5 and 8.0.

Voltage	K_{on} ($M^{-1}s^{-1}$)	K_{off} (s^{-1})	K_D (M^{-1})
+ 25 mV	3.0×10^7	1.6×10^3	5.33×10^{-5}
+ 50 mV	4.0×10^7	2.0×10^3	5.00×10^{-5}
+ 75 mV	5.5×10^7	2.8×10^3	5.09×10^{-5}

Table S2. $Am_6\alpha$CD-binding kinetics with native CymA at pH 8.0

The dissociation constants ($K_D = k_{off}/k_{on}$) were calculated from the association (k_{on}) and dissociation (k_{off}) rate constants. Electrolyte: 1M KCl, 10 mM HEPES, pH 8.0. CDs were added to the trans side. Mean values (± s.d.) from at least three independent experiments (n = 3).

Voltage	K_{on} ($M^{-1}s^{-1}$)	K_{off} (s^{-1})	K_D (M^{-1})
+ 25 mV	1.5×10^7	2.3×10^3	1.53×10^{-4}
+ 50 mV	2.5×10^7	2.8×10^3	1.12×10^{-4}
+ 75 mV	3.5×10^7	4.1×10^3	1.17×10^{-4}

Table S3. $Am_6\alpha$CD-binding kinetics with truncated CymA at pH 8.0

The dissociation constants ($K_D = k_{off}/k_{on}$) were calculated from the association (k_{on}) and dissociation (k_{off}) rate constants. Electrolyte: 1M KCl, 10 mM HEPES, pH 8.0. CDs were added to the trans side. Mean values (± s.d.) from at least three independent experiments (n = 3).

Voltage	K_{on} ($M^{-1}s^{-1}$)	K_{off} (s^{-1})	K_D (M^{-1})
-50 mV	4.0 x 10^5	4.0 x 10^3	1.00 x 10^{-2}
-75 mV	1.0 x 10^6	2.6 x 10^3	2.60 x 10^{-3}
-100 mV	3.1 x 10^6	3.3 x 10^3	1.06 x 10^{-3}
-125 mV	4.5 x 10^6	4.5 x 10^3	1.00 x 10^{-3}

Table S4. $S_6\alpha$CD-binding kinetics with truncated CymA at pH 4.5

The dissociation constants ($K_D = k_{off}/k_{on}$) were calculated from the association (k_{on}) and dissociation (k_{off}) rate constants. Electrolyte: 1M KCl, 10 mM citrate, pH 4.5 CDs were added to the trans side. Mean values (± s.d.) from at least three independent experiments (n = 3).

System	pH	Protein	Cyclodextrin	$q_{Protein}$	q_{CD}
nat-am$_6\alpha$CDpH8.0	8.0	Native CymA	am$_6\alpha$CD	-6	+6
nat-am$_6\alpha$CDpH4.5	4.5	Native CymA	am$_6\alpha$CD	+14	+6
trunc-am$_6\alpha$CDpH8.0	8.0	truncated CymA	am$_6\alpha$CD	-4	+6
trunc-am$_6\alpha$CDpH4.5	4.5	truncated CymA	am$_6\alpha$CD	+15	+6
trunc-s$_6\alpha$CDpH4.5	4.5	truncated CymA	s$_6\alpha$CD	+15	-6
trunc-s$_6\alpha$CDpH8.0	8.0	truncated CymA	s$_6\alpha$CD	-4	-6

Table S5. Description of the different components of the simulation systems: system nomenclatures, pHs, description of the protein, cyclodextrin, charge of the protein and charge of the cyclodextrin. Truncated CymA refers to the CymA protein where residues 1 to 15 were removed. Each simulation system is named after the corresponding cyclodextrin.

System	pH	box-X (nm)	box-Y (nm)	box-Z (nm)	N_{atoms}
nat-am$_6$αCDpH8	8	10.19	10.19	10.91	107345
nat-am$_6$αCDpH4.5	4.5	10.19	10.19	10.91	107304
trunc-am$_6$αCDph4.5	8	10.18	10.18	10.90	107018
trunc-am$_6$αCDpH8	4.5	10.18	10.18	10.90	107069
trunc-s$_6$αCDpH4.5	4.5	10.18	10.18	10.90	107066
trunc-s$_6$αCDpH8	8	10.18	10.18	10.90	106970

Table S6. Description of simulation systems: Simulation system nomenclatures and their corresponding pHs, simulation box vectors in X, Y and Z-directions, and the total number of atoms present in simulation boxes. N_{atoms} refers to the total number of atoms in the simulation systems.

Supplementary Figures

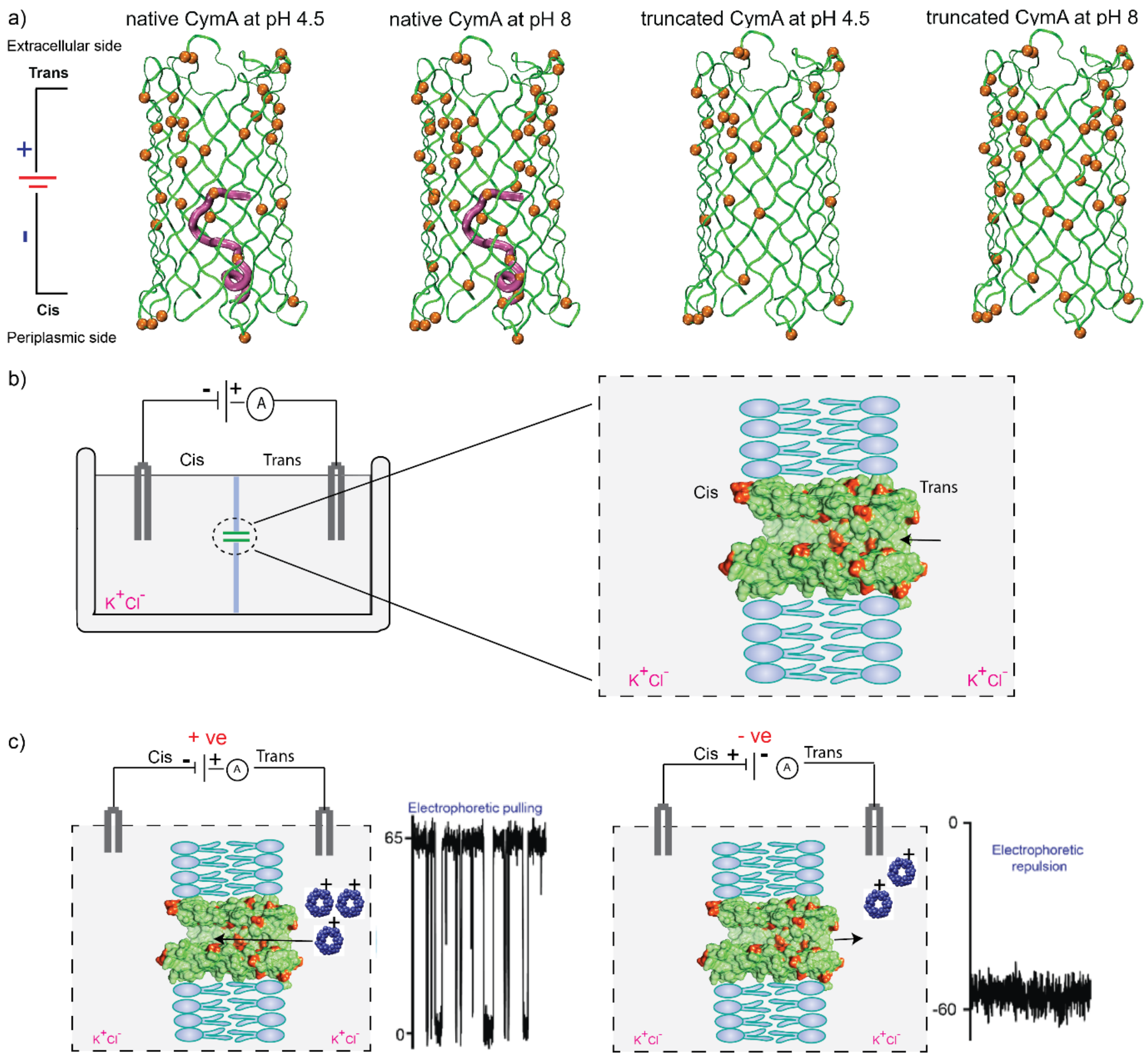

Fig. S1 CymA structures, orientation and electrophoretic pulling of cationic analytes.

a) The pores are shown in a ribbon representation (green), and the negatively charged amino acids are shown in a space-filling sphere representation (orange). The N terminus segment is shown by a tube representation (red). **b)** A schematic showing pore insertion and orientation in the planar lipid bilayers. The extracellular part of the pore composed of dense negatively charged residues is exposed to the trans compartment. **c)** The voltage-driven electrophoretic pulling of cationic analytes (represented in green) added to the trans side into the pore, specifically at positive voltages, resulting in ion current blockages. No blockages are observed at negative voltages due to electrophoretic repulsion of cationic analytes added to the trans side of the pore.

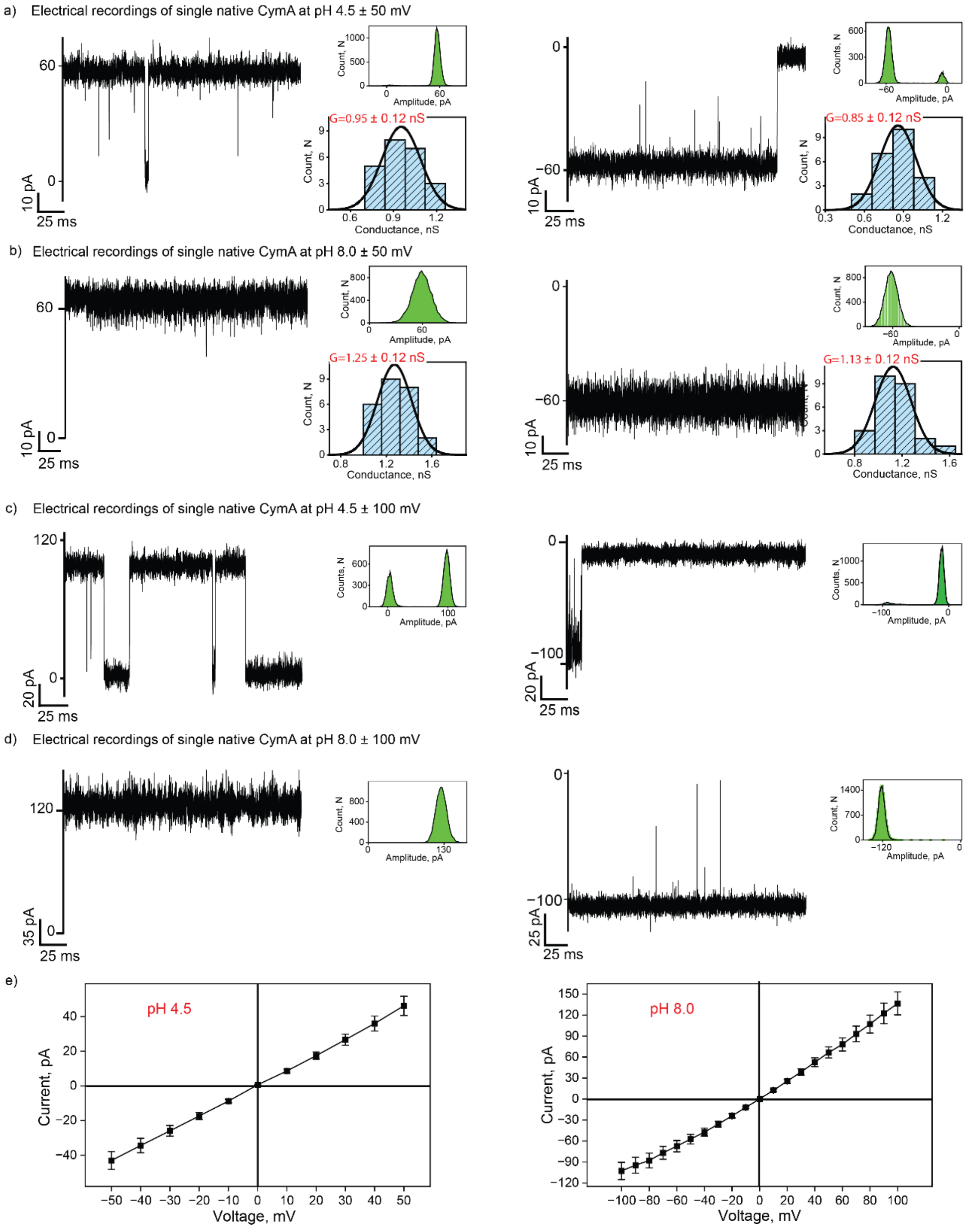

Fig. S2 Gating pattern of native CymA.

a) Electrical recordings of single native CymA at ±50 mV in pH 4.5. The unitary conductance (n=25) and current amplitude histogram are shown. **b)** Electrical recordings of single native CymA at ±50 mV in pH 8.0. The unitary conductance (n=25) and current amplitude histogram are shown. **c)** Electrical recordings of single native CymA at ±100 mV in pH 4.5 and **d)** pH 8.0. All points current amplitude histogram is shown. **e)** I-V curve obtained from a single native CymA in pH 4.5 and 8.0. The current signals were digitally filtered at 7 kHz.

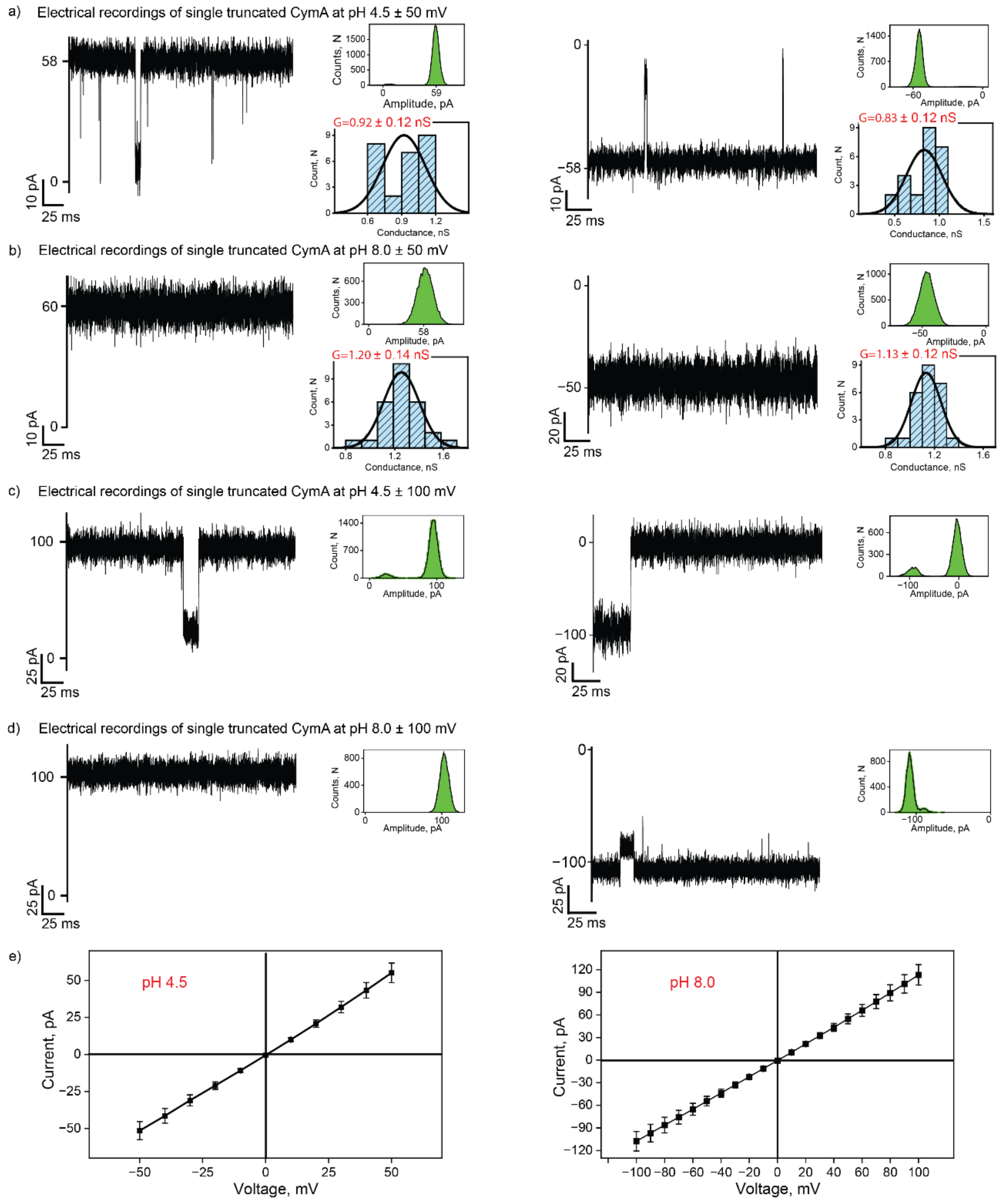

Fig. S3 Gating pattern of truncated CymA.

a) Electrical recordings of single truncated CymA at ±50 mV in pH 4.5. The unitary conductance (n=25) and current amplitude histogram are shown. **b)** Electrical recordings of single truncated CymA at ±50 mV in pH 8.0. The unitary conductance (n=25) and current amplitude histogram are shown. **c)** Electrical recordings of single truncated CymA at ±100 mV in pH 4.5 and current amplitude histogram are shown. **d)** Electrical recordings of single truncated CymA at ±100 mV in pH 8.0 and current amplitude histogram are shown. **e)** I-V curve obtained from a single truncated CymA in pH 4.5 and 8.0. The current signals were digitally filtered at 7 kHz.

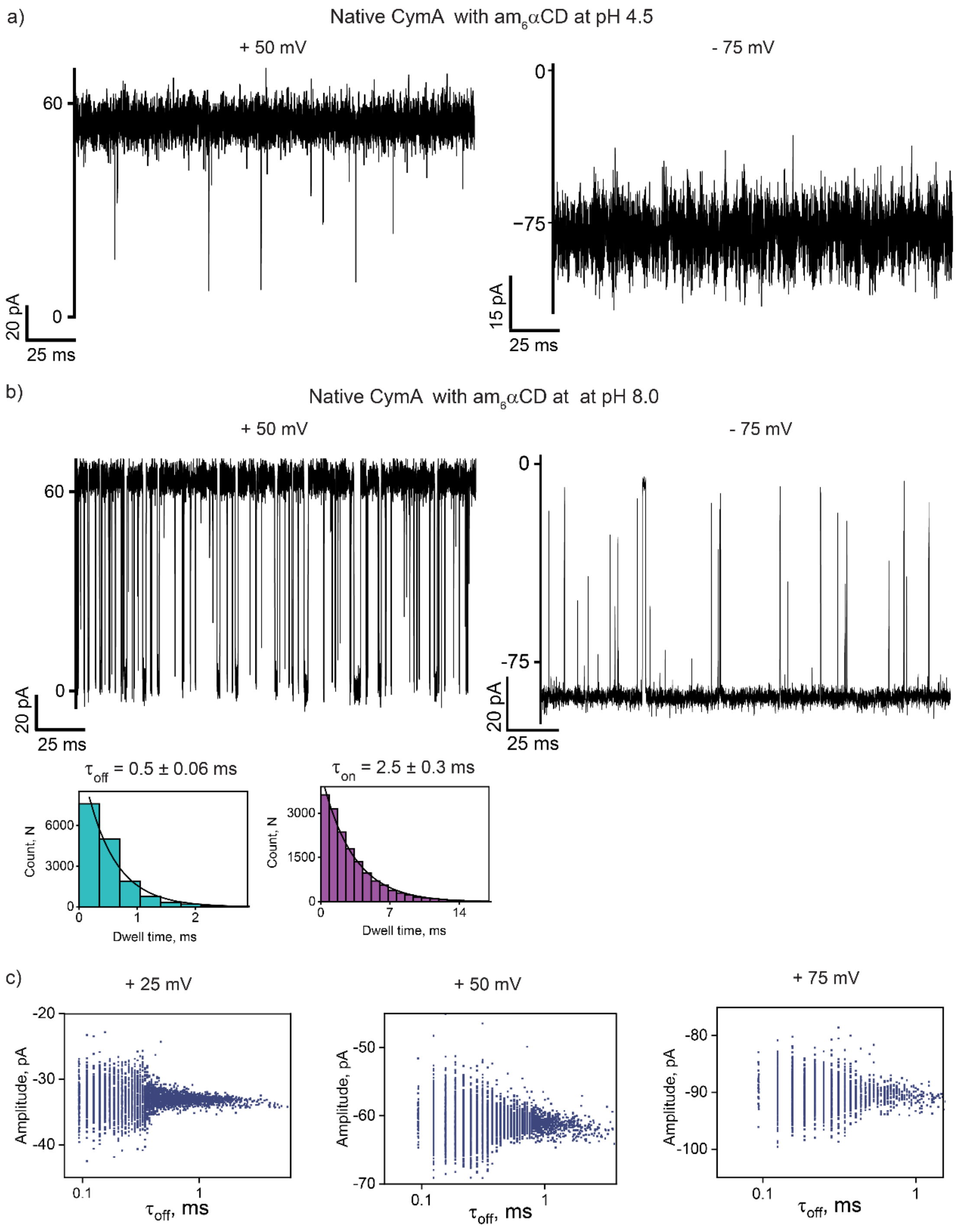

Fig. S4 Interaction of am$_6\alpha$CD with native CymA under different pH conditions.

a) Electrical recordings of single native CymA in the presence of am$_6\alpha$CD (10 µM, trans) at +50 mV and -75 mV in pH 4.5 **b)** Electrical recordings of single native CymA in the presence of am$_6\alpha$CD (10 µM, trans) at +50 mV and -75 mV in pH 8.0. Insets show the corresponding τ_{off} and τ_{on} dwell time histograms of CD blocking fitted with a monoexponential probability function. **c)** Scatter plots of current block amplitudes versus dwell time (τ_{off}) of CD blocking with native CymA are shown. The current signals were digitally filtered at 7 kHz.

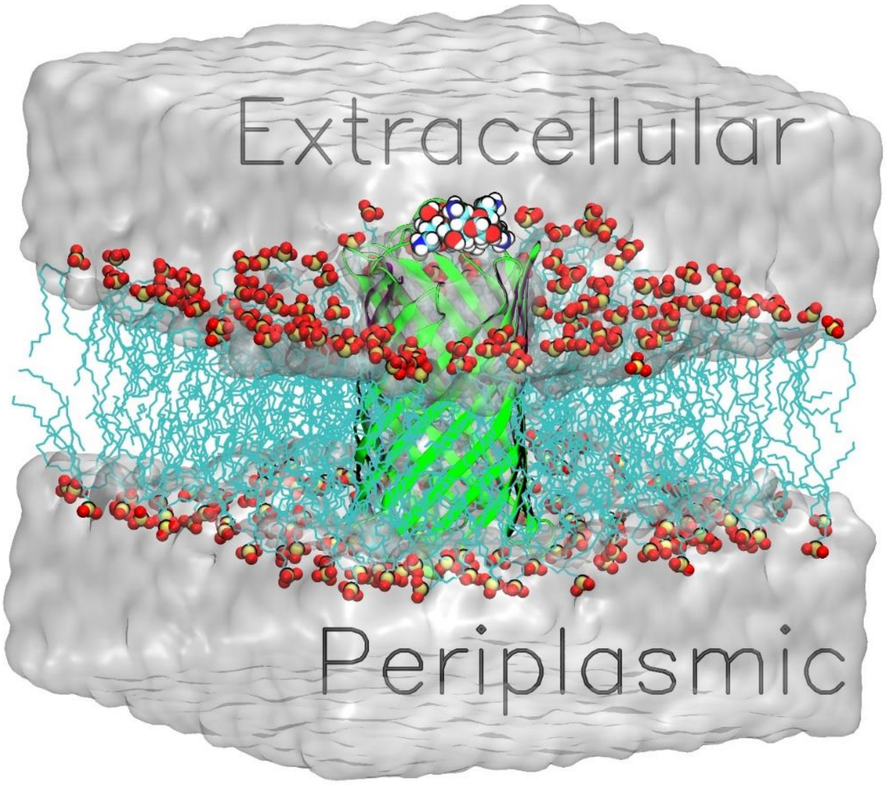

Fig. S5 Representative configuration of the hydrated CymA embedded in DPhPC lipid bilayer MD-simulation system with am$_6\alpha$CD.

The water layer on top and bottom of the lipid bilayer is shown as gray transparent slabs. The hydrophobic chains of the lipids are shown in cyan color. The phosphate groups of the lipids are shown as vdW spheres where the P-atom is shown in yellow and the O-atom in red colored spheres. The native CymA is shown in the green cartoon representation. The am$_6\alpha$CD is shown using vdW sphere representation where the C-atoms are shown as cyan spheres, H-atoms as white and O-atoms as red-colored spheres.

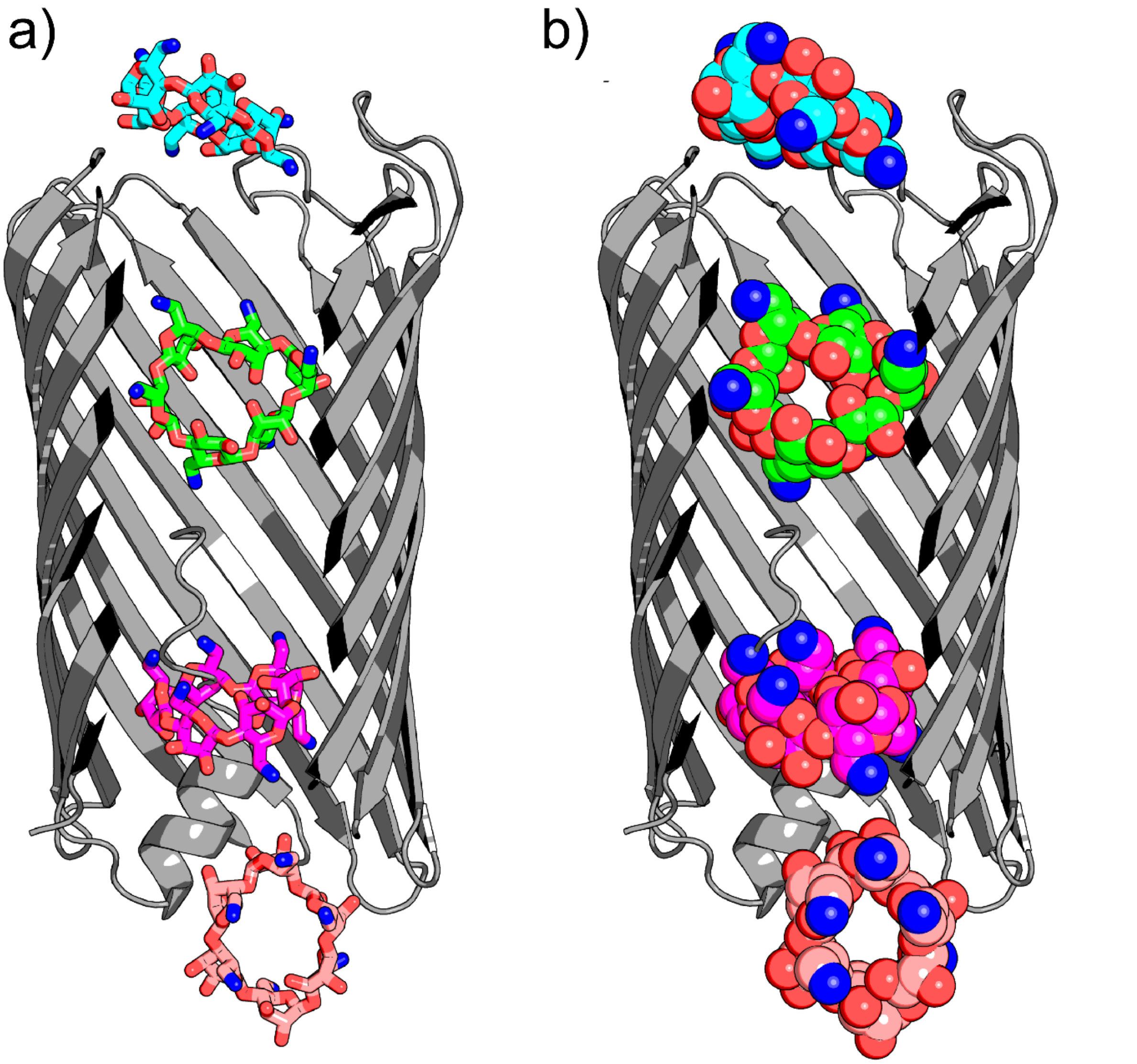

Fig. S6 Multiple configurations of the am$_6$αCD while translocating through the native CymA. The CD assumes different configurations from the extracellular side's entrance to the periplasmic side's exit. The native CymA pore is shown in a cartoon (gray) representation. In **a)** The CD was shown using a stick representation, and the C-atoms were colored differently, rendering different conformations of the CD at various stages of the translocation event. In **b)** CD configurations with space-filling sphere representations for visual comparison.

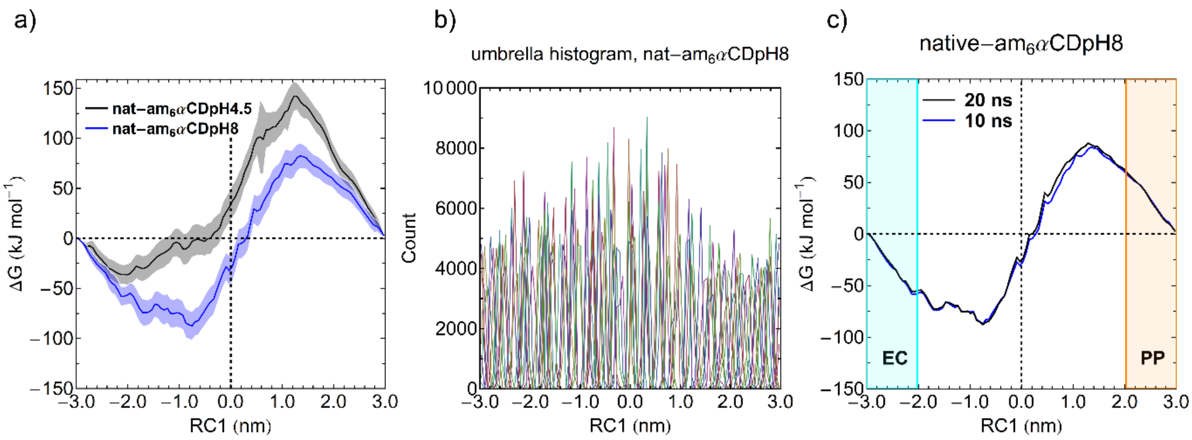

Fig. S7 A one-dimensional free energy profile was obtained for am₆αCD translocation through native CymA at pH 4.5 and 8.0.

a) The error bars associated with the free energy calculation are calculated using a bootstrapping method implemented in gmx wham utility of GROMACS MD simulation software. The error bars are black (pH 4.5) and blue (pH 8.0) transparent filling on the respective free energy profiles. **b)** The umbrella histogram (121 windows) overlaps for native CymA umbrella sampling simulations at pH 8.0 for RC1 ranging from -3 to +3 nm. **c)** The free energy profiles for am₆αCD translocation through native CymA at pH 8.0 obtained from 10 ns (blue) and 20 ns (black) simulation times for 121 umbrella windows spanning a range of -3.0 to +3.0 nm for the z-component of protein-ligand center-to-center distances or RC1.

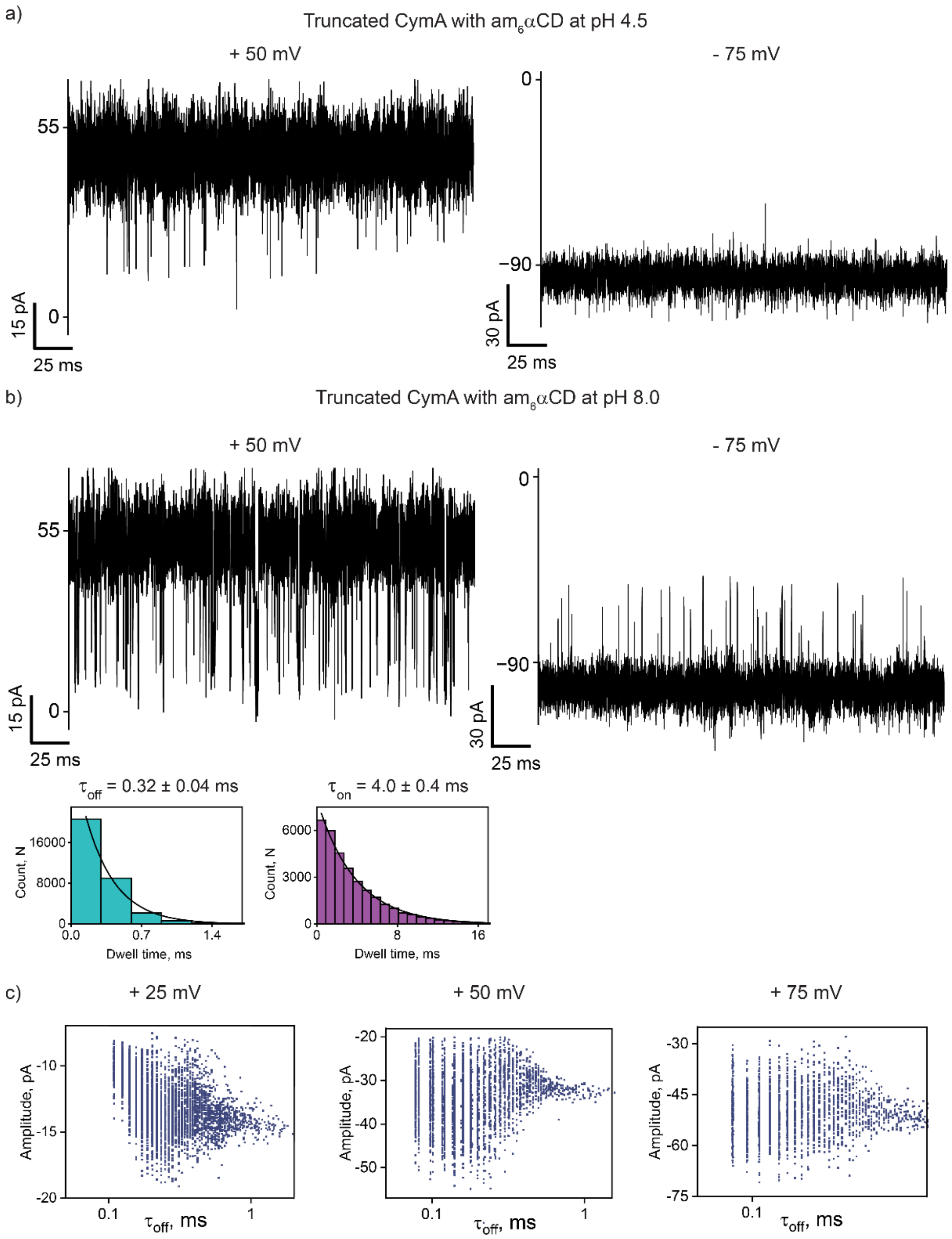

Fig. S8 Interaction of am$_6\alpha$CD with truncated CymA at pH 4.5 and 8.0.

a) Electrical recordings of single truncated CymA in the presence of am$_6\alpha$CD (10 µM, trans) at +50 mV and -75 mV in pH 4.5 **b)** Electrical recordings of single truncated CymA in the presence of am$_6\alpha$CD (10 µM, trans) at +50 mV and -75 mV in pH 8.0. Insets show the corresponding τ_{off} and τ_{on} dwell time histograms of CD blocking fitted with a monoexponential probability function. **c)** Scatter plots of current block amplitudes versus dwell time (τ_{off}) of CD blocking truncated CymA are shown. The current signals were digitally filtered at 7 kHz.

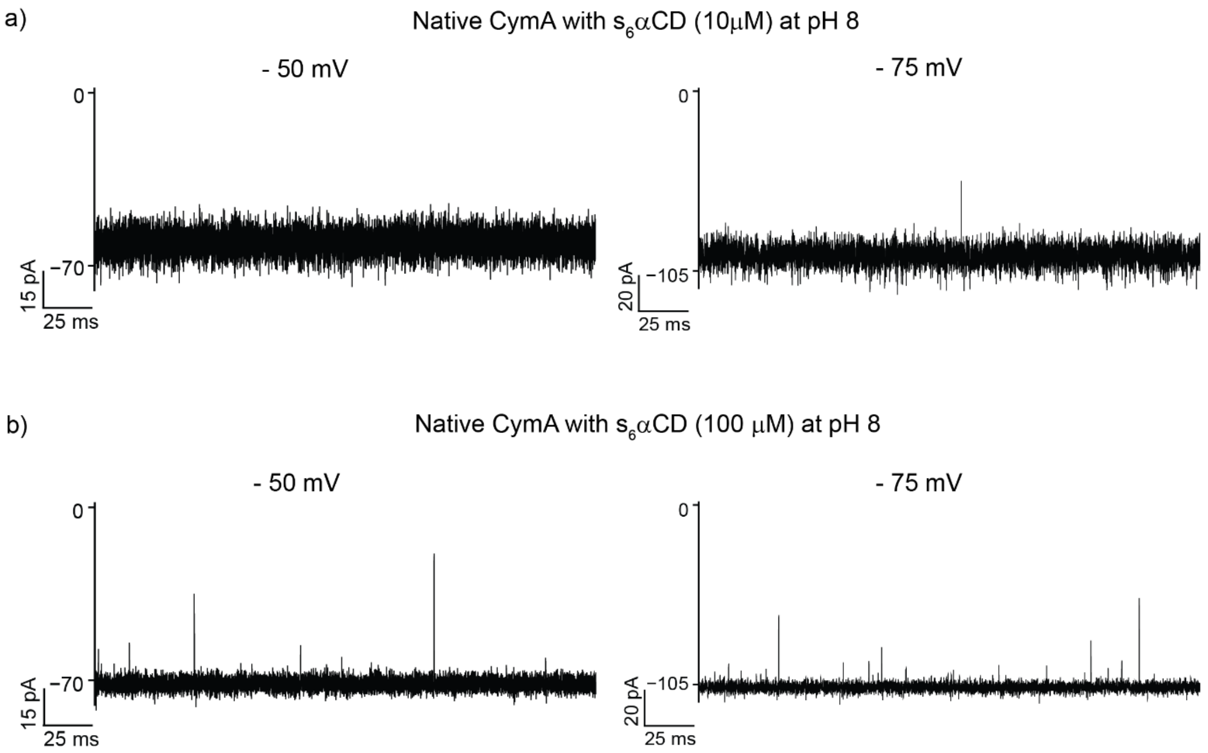

Fig. S9 Interaction of s6αCD with native CymA under different pH conditions.

a) Electrical recordings of single native CymA in the presence of s6αCD (10 µM, trans) at -50 mV and - 75 mV in pH 8.0 **b)** Electrical recordings of single native CymA in the presence of s6αCD (100 µM, trans) at -50 mV and -75 mV at pH 8.0. The current signals were digitally filtered at 7 kHz.

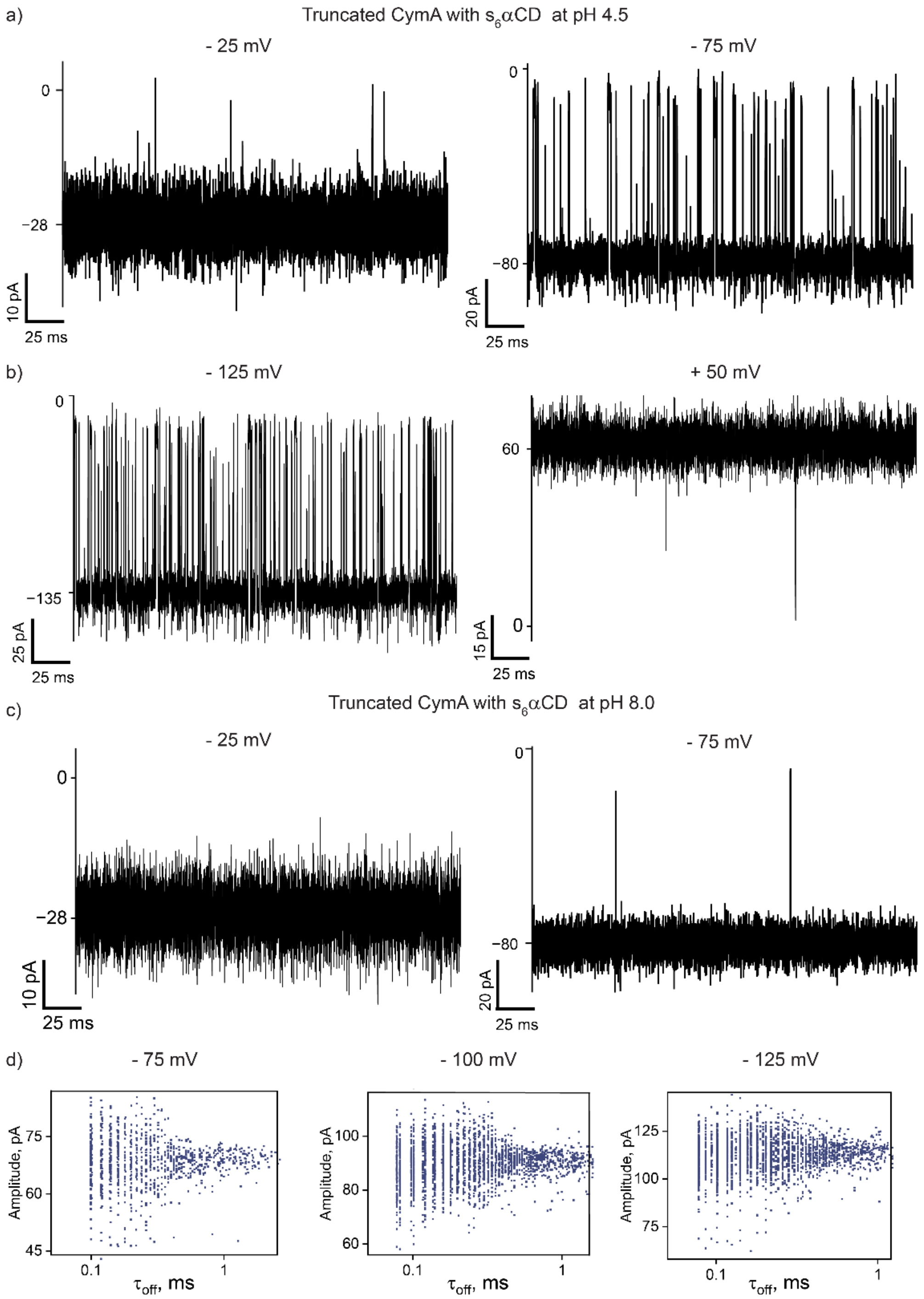

Fig. S10 Interaction of s6αCD with truncated CymA under different pH conditions.

a) Electrical recordings of single truncated CymA in the presence of s6αCD (100 µM, trans) at -25 mV and -75 mV in pH 4.5 **b)** -125 mV and +50 mV in pH 4.5. **c)** Electrical recordings of single truncated CymA in the presence of s6αCD (100 µM, trans) at -25 mV and -75 mV in pH 8.0. **c)** Scatter plots of current block amplitudes versus dwell time (τ_{off}) of CD blocking truncated CymA are shown. The current signals were digitally filtered at 7 kHz.

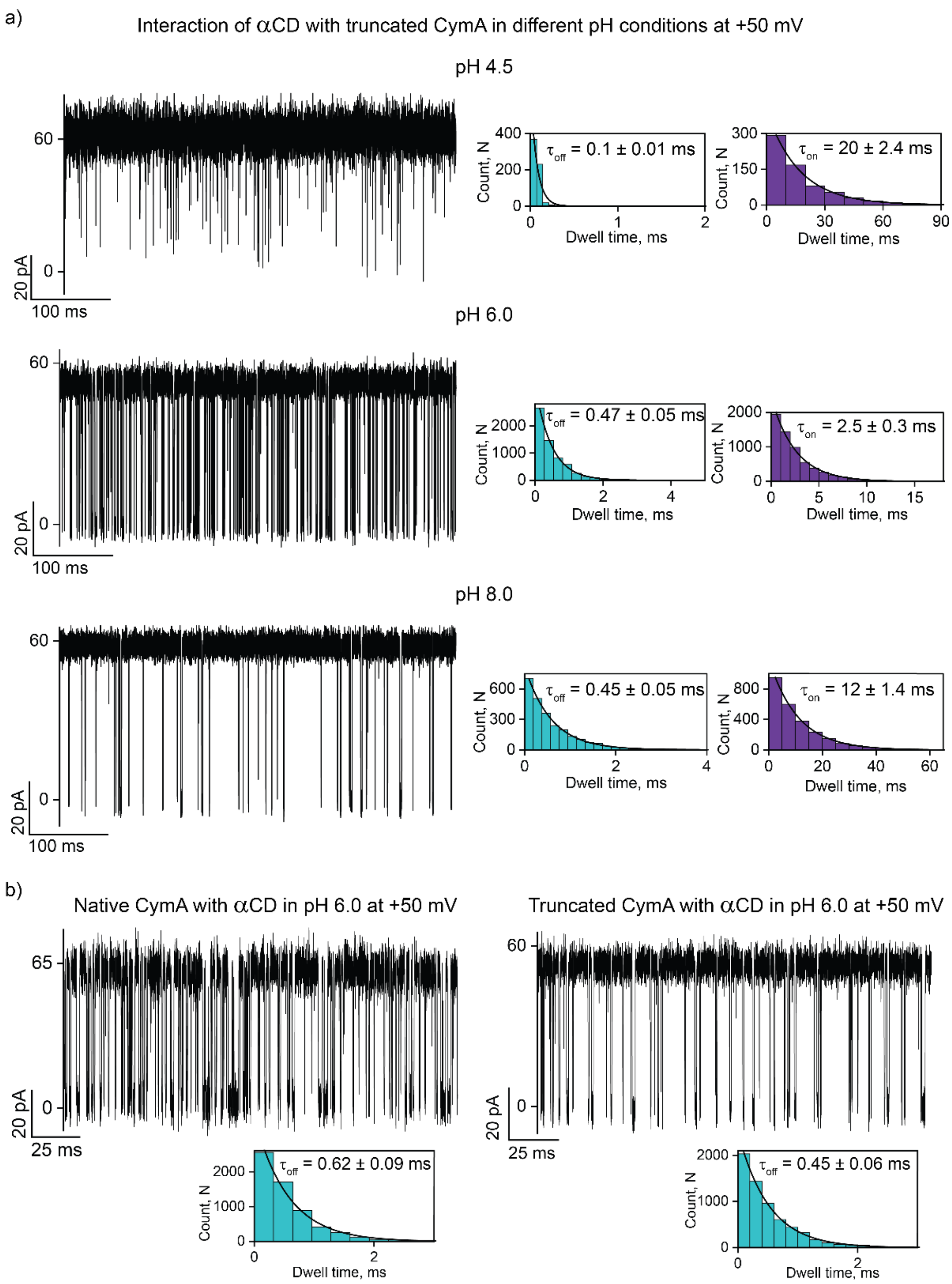

Fig. S11 Interaction of neutral αCD with CymA pores at different pHs.

a) Electrical recordings of single truncated CymA in the presence of αCD (100 µM, trans) at +50 mV in pH 4.5. Electrical recordings of single truncated CymA in the presence of αCD (10 µM, trans) at +50 mV in pH 6.0 and pH 8.0. Insets show the corresponding τ_{off} and τ_{on} dwell time histograms of CD blocking fitted with a monoexponential probability function. **b)** Electrical recordings of a single native and truncated CymA in the presence of neutral αCD (10 µM, trans) at +50 mV in pH 6.0. The current signals were digitally filtered at 7 kHz.

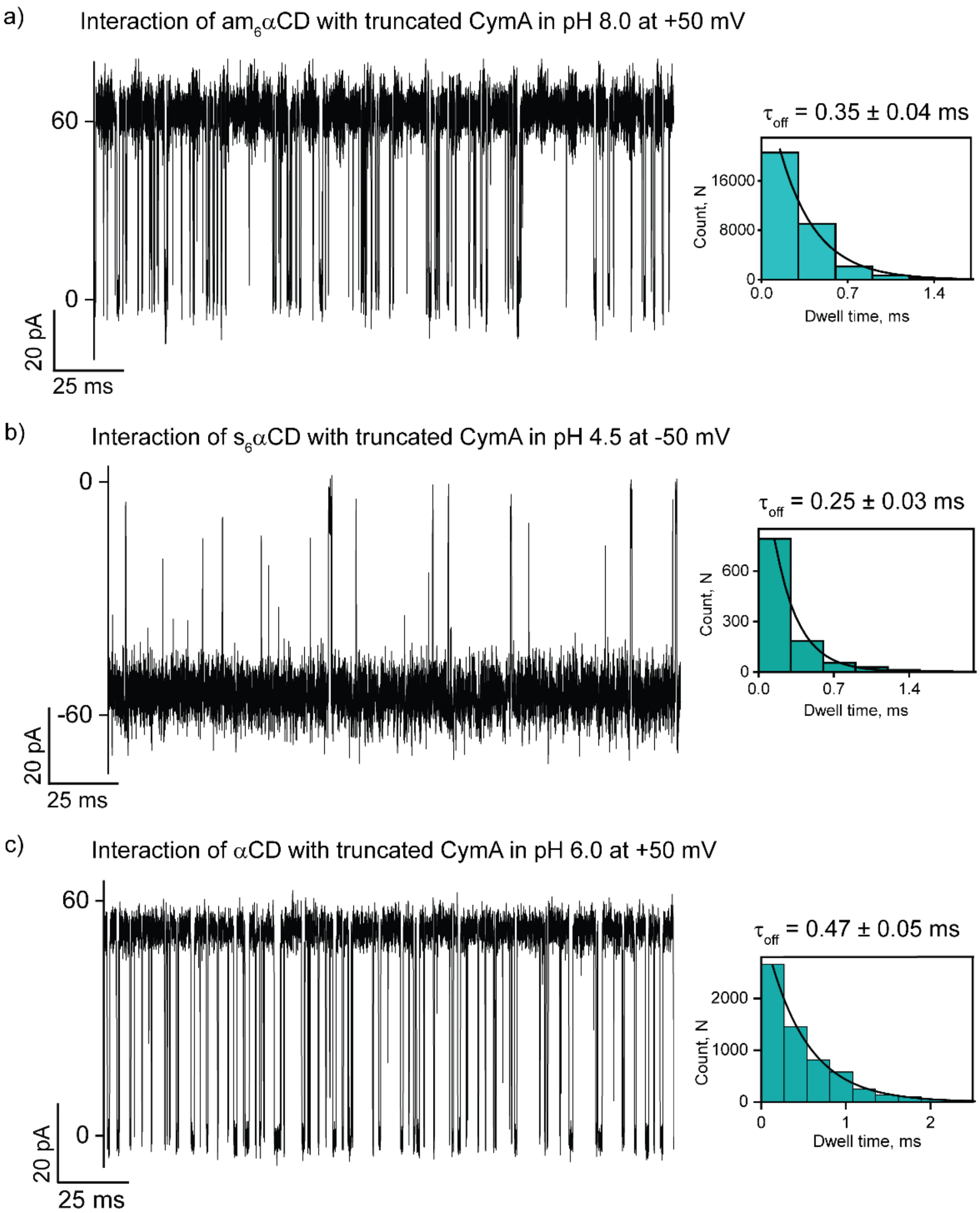

Fig. S12 Interaction of am$_6\alpha$CD, s$_6\alpha$CD and αCD with truncated CymA.

a) Electrical recordings of single truncated CymA in the presence of am$_6\alpha$CD (10 µM, trans) at +50 mV in pH 8.0 **b)** Electrical recordings of single truncated CymA in the presence of s$_6\alpha$CD (100 µM, trans) at -50 mV in pH 4.5. **c)** Electrical recordings of single truncated CymA in the presence of neutral αCD (10 µM, trans) at +50 mV in pH 6.0. The current signals were digitally filtered at 7 kHz.

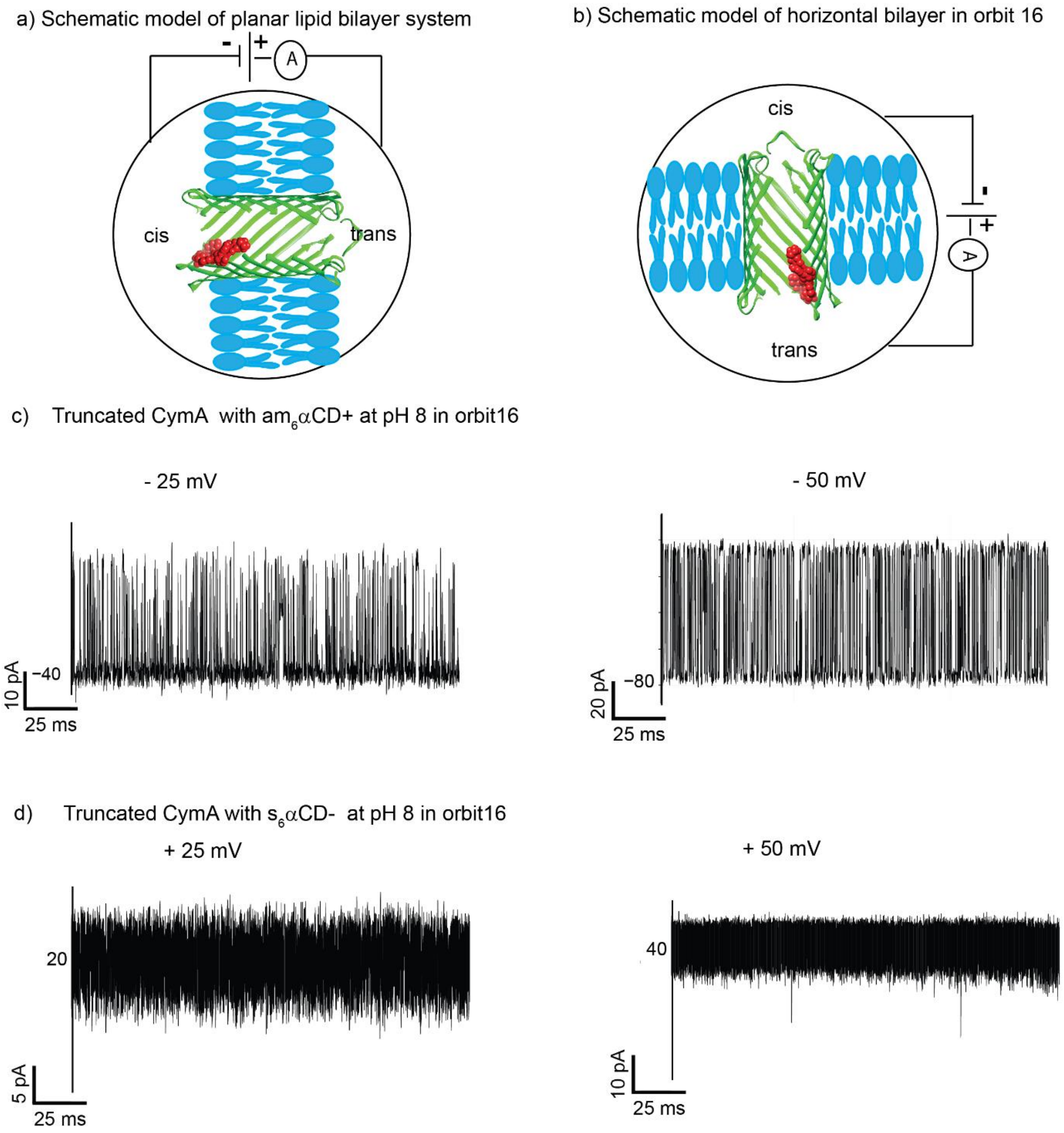

Fig. S13 Interaction of am$_6$αCD and s$_6$αCD with truncated CymA at pH 8 in orbit16.

a) Schematic of CymA pore orientation in planar lipid bilayer system. **b)** Schematic of CymA pore orientation in Orbit 16 system. **c)** Electrical recordings of single truncated CymA in the presence of am$_6$αCD (10 µM, cis) at -25 mV and - 50 mV in pH 8.0. **d)** Electrical recordings of single truncated CymA in the presence of s$_6$αCD (100 µM) at +25 mV and +50 mV in pH 8.0. The current signals were filtered at 10 kHz and sampled at 20 kHz.

a) Crystal structure of native CymA b) Modelled structure of native CymA c) Modelled structure of native CymA in the presence of cyclodextrin

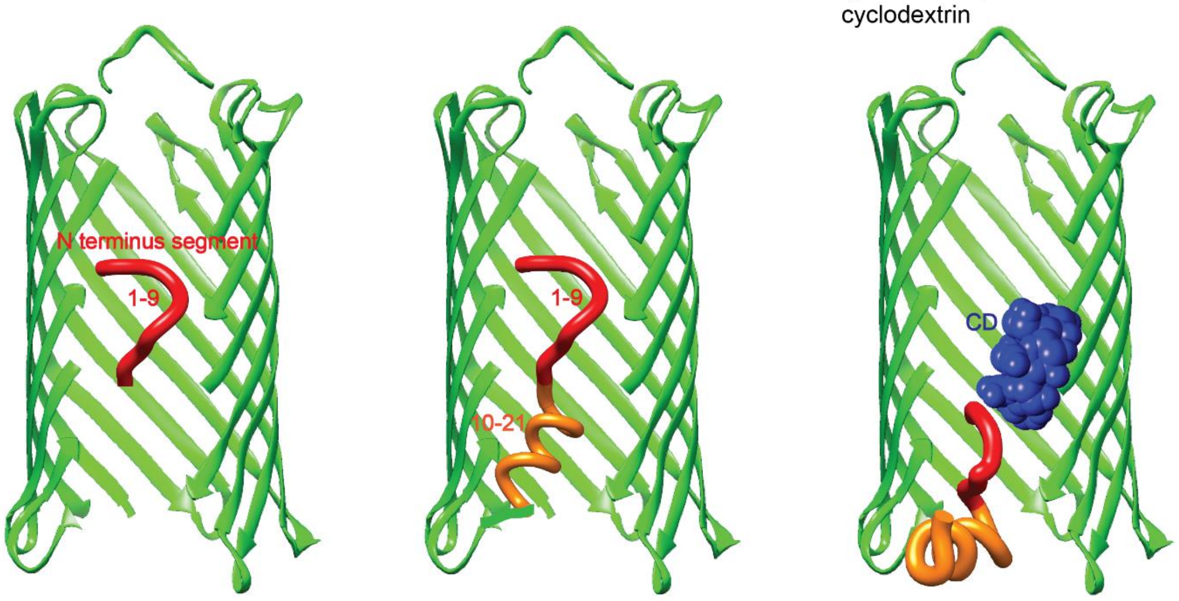

Fig. S14 Molecular model of substrate translocation through CymA.

a) Crystal structure of native CymA highlighting the presence of resolved N terminus segment (residues 1-9). **b)** Modelled native CymA highlighting the entire N terminus segment in the pore. **c)** Translocation of am$_6\alpha$CD across native CymA and associated N terminus segment conformational dynamics.

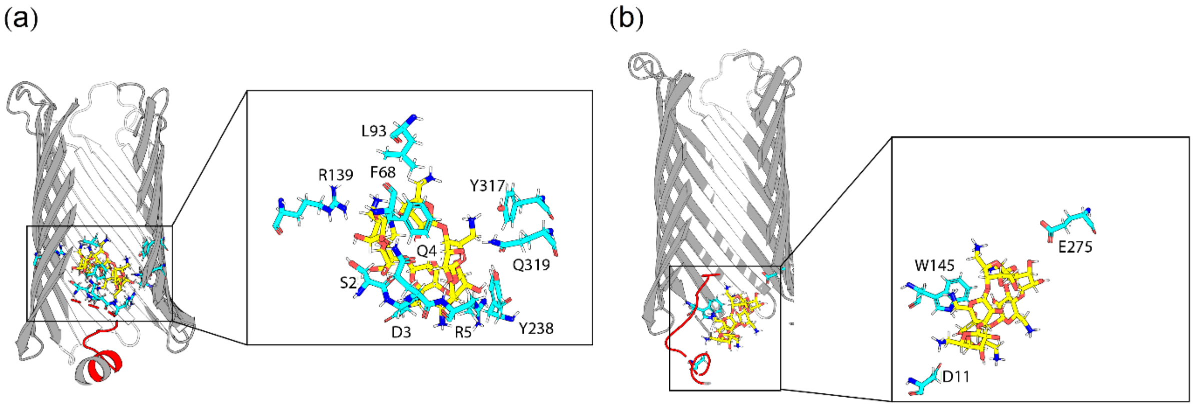

Fig. S15 CymA protein residues involved in polar interactions with am$_6\alpha$CD

a) amino acid residues involved in polar interactions with the ligand where the Nterm loop and the ligand coexist inside the pore when the ligand crosses the pore's center. The configuration refers to the Fig. 6C snapshot in the main text. **b)** amino acid residues involved in polar interactions with the ligand where the Nterm loop is inside while the ligand has translocated almost outside the pore towards the periplasmic region. The configuration refers to the Fig. 6D snapshot. The protein is shown in cartoon representation (grey), the N-term loop region (residue index 1-15) is shown in cartoon representation (red) and the amino acid residues involved in polar interactions are shown as sticks (C-cyan, O-red, N-blue, H-white) and the am$_6\alpha$CD ligand is shown in sticks representation (C-yellow, O-red, H-white)

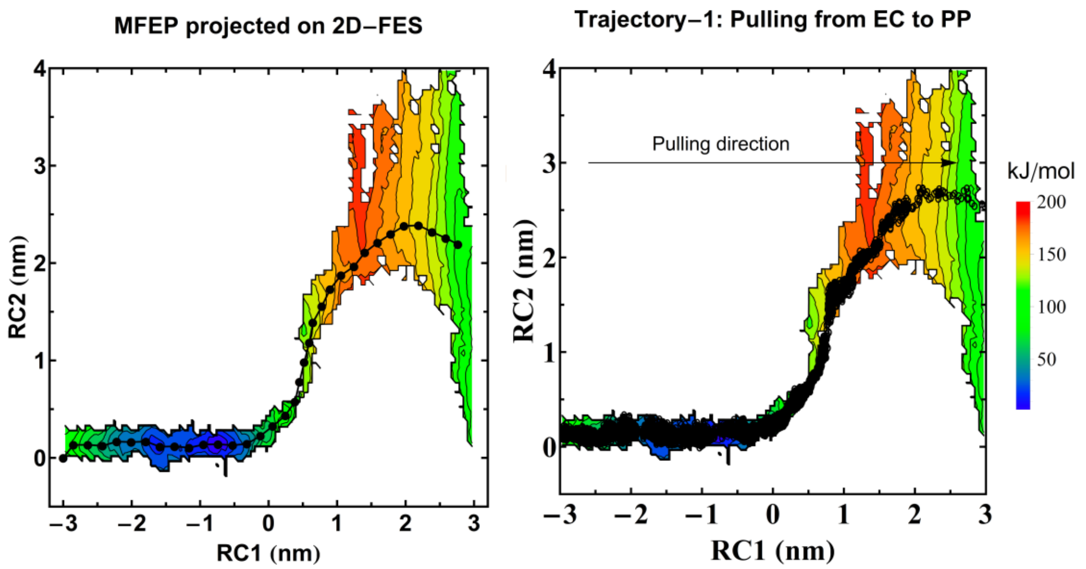

Fig. S16 A minimum free energy path (MFEP) obtained using a post-string approach. (MFEP) obtained using a post-string approach described by Morita et al[17] and the steered MD trajectory is projected onto the 2D-FES as a function of RC1 and RC2, for am$_6\alpha$CD translocation through native CymA at pH 8.

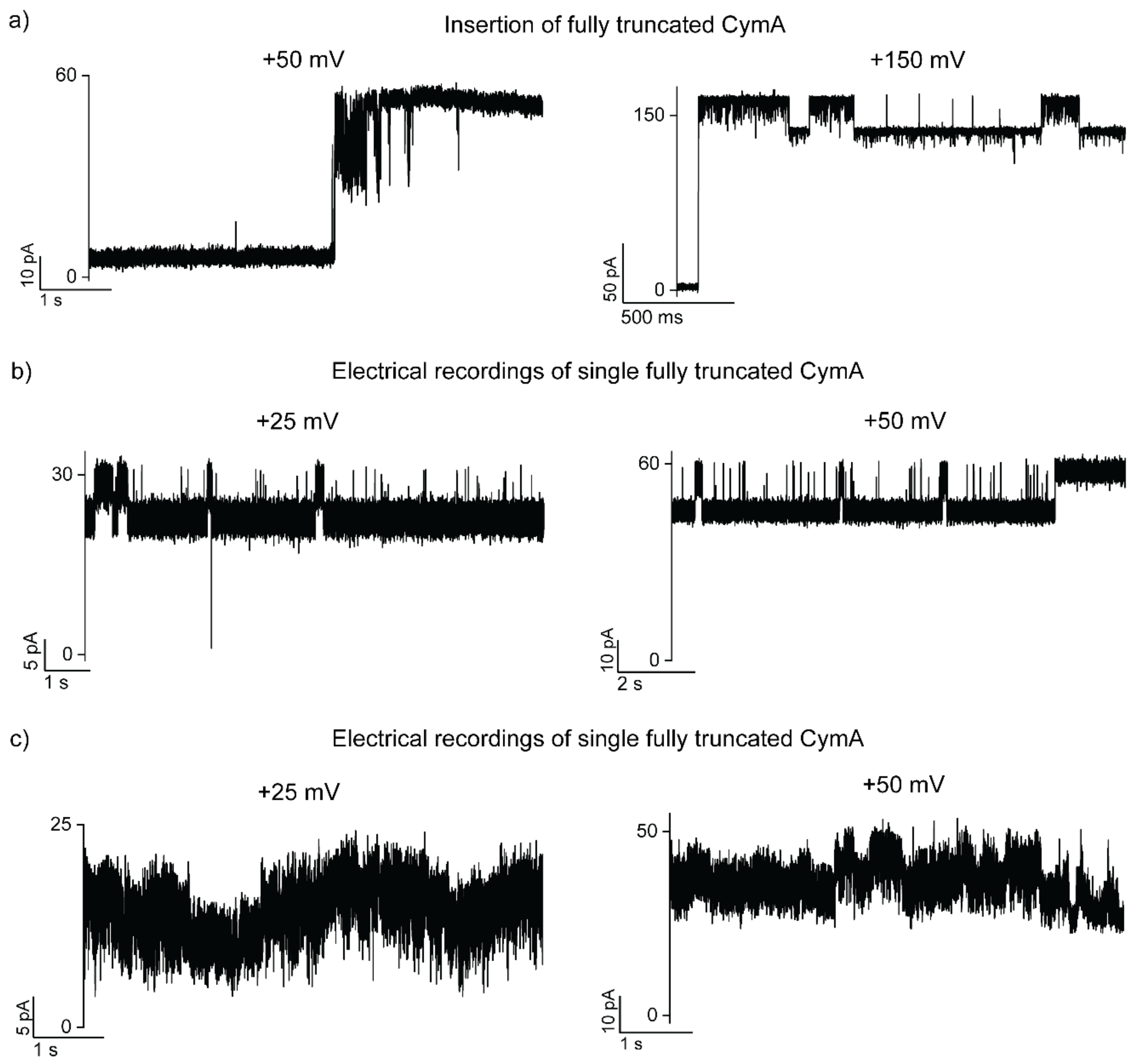

Fig. S17 Single-channel properties of fully truncated CymA.

a) Electrical recordings of fully truncated CymA insertion at +50 mV and +150 mV in pH 8.0. **b)** Electrical recordings of fully truncated single CymA at +25 mV and +50 mV showing sub conductance states in pH 8.0. **c)** Electrical recordings of fully truncated single CymA showing unstable states at +25 mV and +50 mV in pH 8.0. The current signals were filtered at 2 kHz and sampled at 10 kHz.

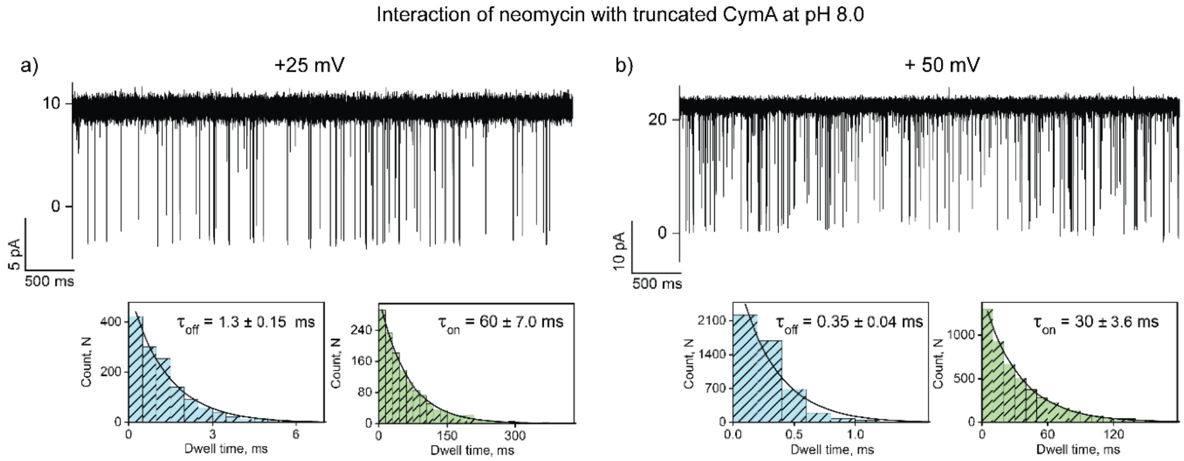

Fig. S18 Interaction of neomycin with truncated CymA.

a) Electrical recordings of single truncated CymA in the presence of neomycin (10 µM, trans) at +25 mV. **b)** +50 mV in pH 8. Insets show the corresponding τ_{off} and τ_{on} dwell time histograms of neomycin blocking fitted with a monoexponential probability function. The current signals were digitally filtered at 7 kHz.

References:

1. S. Jo, T. Kim, V. G. Iyer and W. Im, *J. Comput. Chem.*, 2008, **29**, 1859-1865.

2. T. J. Dolinsky, P. Czodrowski, H. Li, J. E. Nielsen, J. H. Jensen, G. Klebe and N. A. Baker, *Nucleic Acids Research.*, 2007, **35**, W522-525.

3. M. H. Olsson, C. R. Sondergaard, M. Rostkowski and J. H. Jensen, *J. Chem. Theory Comput.*, 2011, **7**, 525-537.

4. A. D. MacKerell, D. Bashford, M. Bellott, R. L. Dunbrack, J. D. Evanseck, M. J. Field, S. Fischer, J. Gao, H. Guo, S. Ha, D. Joseph-McCarthy, L. Kuchnir, K. Kuczera, F. T. Lau, C. Mattos, S. Michnick, T. Ngo, D. T. Nguyen, B. Prodhom, W. E. Reiher, B. Roux, M. Schlenkrich, J. C. Smith, R. Stote, J. Straub, M. Watanabe, J. Wiorkiewicz-Kuczera, D. Yin and M. Karplus, *J. Phys.Chem. B.*, 1998, **102**, 3586-3616.

5. J. B. Klauda, R. M. Venable, J. A. Freites, J. W. O'Connor, D. J. Tobias, C. Mondragon-Ramirez, I. Vorobyov, A. D. MacKerell, Jr. and R. W. Pastor, *J. Phys. Chem. B.*, 2010, **114**, 7830-7843.

6. K. Vanommeslaeghe, E. Hatcher, C. Acharya, S. Kundu, S. Zhong, J. Shim, E. Darian, O. Guvench, P. Lopes, I. Vorobyov and A. D. Mackerell, Jr., *J. Comput. Chem.*, 2010, **31**, 671-690.

7. R. B. Best, X. Zhu, J. Shim, P. E. Lopes, J. Mittal, M. Feig and A. D. Mackerell, Jr., *J. Chem. Theory Comput.*, 2012, **8**, 3257-3273.

8. S. Páll and B. Hess, *Comput. Phys. Commun.*, 2013, **184**, 2641-2650.

9. T. Darden, D. York and L. Pedersen, *J. Chem. Phys.*, 1993, **98**, 10089-10092.

10. B. Hess, H. Bekker, H. J. C. Berendsen and J. G. E. M. Fraaije, *J. Comput. Chem.*, 1997, **18**, 1463-1472.

11. S. Miyamoto and P. A. Kollman, *J. Comput. Chem.*, 1992, **13**, 952-962.

12. H. J. C. Berendsen, J. P. M. Postma, W. F. van Gunsteren, A. DiNola and J. R. Haak, *J. Chem. Phys.*, 1984, **81**, 3684-3690.

13. S. Nosé, *Mol. Phys.*, 1984, **52**, 255-268.

14. M. Parrinello and A. Rahman, *J. Appl. Phys.*, 1981, **52**, 7182-7190.

15. J. S. Hub, B. L. de Groot and D. van der Spoel, *J. Chem. Theory Comput.*, 2010, **6,** 3713-3720.

16. W. Humphrey, A. Dalke and K. Schulten, *J.Mol. Graph.*, 1996, **14**, 33-38, 27-38.

17. R. Morita, Y. Shigeta and R. Harada, *Chem. Phys. Lett.*, 2021, **782**, 139003.
